# An integrative phenology and climatic suitability model for emerald ash borer

**DOI:** 10.3389/finsc.2023.1239173

**Published:** 2023-08-29

**Authors:** Brittany S. Barker, Leonard Coop, Jian J. Duan, Toby R. Petrice

**Affiliations:** ^1^ Oregon Integrated Pest Management Center, Oregon State University, Corvallis, OR, United States; ^2^ Department of Horticulture, Oregon State University, Oregon State University, Corvallis, OR, United States; ^3^ United States Department of Agriculture (USDA) Agricultural Research Service, Beneficial Insects Introduction Research Unit, Newark, DE, United States; ^4^ United States Department of Agriculture (USDA) Forest Service, Northern Research Station, Lansing, MI, United States

**Keywords:** invasive species, *Agrilus planipennis*, *Fraxinus*, forecast, surveillance, management, thermal stresses

## Abstract

**Introduction:**

Decision support models that predict both when and where to expect emerald ash borer (EAB), *Agrilus planipennis* Fairmaire (Coleoptera: Buprestidae), are needed for the development and implementation of effective management strategies against this major invasive pest of ash (*Fraxinus* species) in North America and other regions such as Europe. We present a spatialized model of phenology and climatic suitability for EAB for use in the Degree-Days, Risk, and Phenological event mapping (DDRP) platform, which is an open-source decision support tool to help detect, monitor, and manage invasive threats.

**Methods:**

We evaluated the model using presence records from three geographic regions (China, North America, and Europe) and a phenological dataset consisting primarily of observations from the northeastern and midwestern United States. To demonstrate the model, we produced phenological event maps for a recent year and tested for trends in EAB’s phenology and potential distribution over a recent 20-year period.

**Results:**

Overall, the model exhibited strong performance. Presence was correctly estimated for over 99% of presence records and predicted dates of adult phenological events corresponded closely with observed dates, with a mean absolute error of *ca.* 7 days and low estimates of bias. Climate stresses were insufficient to exclude EAB from areas with native *Fraxinus* species in North America and Europe; however, extreme weather events, climate warming, and an inability for EAB to complete its life cycle may reduce suitability for some areas. Significant trends toward earlier adult emergence over 20 years occurred in only some areas.

**Discussion:**

Near real-time model forecasts for the conterminous United States are available at two websites to provide end-users with decision-support for surveillance and management of this invasive pest. Forecasts of adult emergence and egg hatch are particularly relevant for surveillance and for managing existing populations with pesticide treatments and parasitoid introductions.

## 1 Introduction

Invasive insects are one of the most serious and urgent ecological and economic threats to forests in North America and Europe (1, 2). Phenological maps may increase the effectiveness of eradication or management of invasive insect species because knowing where and when to expect a specific life stage may increase the likelihood of pest detection (1–4) and improve the timing of control measures against the susceptible life stage (5, 6). Likewise, maps of establishment risk can help decision-makers know whether to invest in pre-arrival strategies, such as surveillance and attaining approval for tree removal, chemical treatments, and biological control, to mitigate against the forest pest’s colonization and establishment (7, 8).

Native to East Asia, the emerald ash borer (EAB), *Agrilus planipennis* Fairmaire (Coleoptera: Buprestidae) is a major threat to ash species (*Fraxinus* species, Oleaceae) in North America and Europe, all of which are susceptible to this wood-boring beetle to at least some extent (9–12). In North America, EAB was first detected in Michigan in 2002 and is now present in 36 U.S. states and the District of Columbia, in addition to five Canadian Provinces (Ontario, Quebec, New Brunswick, Novia Scotia and Manitoba) (13–17). In Europe, the pest was first found in Moscow in 2003 and has since spread throughout European Russia and eastern Ukraine (18–21). Ash declines caused by EAB have had devastating impacts on ecosystem processes, ecological services, sociocultural practices, and local and regional economies (15, 22–25). These impacts will likely grow if EAB continues its expansion into new areas such as the Pacific Coast of the United States (26) and Central and Western Europe (7, 12, 27, 28). Spatial forecasts of phenology and establishment risk are urgently needed for managing infestations and slowing the spread of invasive EAB populations (3, 7, 12).

Phenology models that predict the seasonal timing of EAB’s life cycle can help decision-makers know when to install surveillance equipment and implement control tactics that target a specific life stage (5). EAB is extremely difficult to detect in part because immature life stages develop underneath tree bark (29, 30). Forecasts of phenology for the adult stage, which occurs outside of trees, may help detect newly established and low density populations, potentially providing time to reduce adult dispersal to new locations (15, 31). Additionally, forecasts of adult activity and egg hatch can help properly time the application of systemic insecticides (5) and the release of parasitoids that target EAB eggs or larvae (32–35). Published phenology models developed for EAB include those that predict adult emergence (5, 36, 37), first oviposition (32), and the proportion of insects overwintering as J-larvae (33). However, only one model was operationalized to produce regularly updated forecasts of adult activities based on degree-days, which made it useful for real-time decision support (5, 38). This model was developed from adult EAB trap captures primarily from the midwestern United States and its performance has not been evaluated.

Several studies have assessed establishment risk for EAB based on climate, which is a regional-scale indicator of environmental suitability for organisms (8, 39, 40). Climatic suitability models for EAB generally indicate that most or all of the ranges of native *Fraxinus* species in North America and Europe are suitable for establishment (41–48). However, most of these models used climate normals (41–47) or climate data for a past year (48), which provides only a single snapshot of establishment risk and may not reflect present-day suitability because of rapid climate change in both continents (49–53). Indeed, one model predicted reductions in overwintering mortality in EAB in North America between 1973 and 2013, which suggests that suitability has increased in some areas that were previously too cold for establishment (45). Additionally, climate normals may essentially erase signals of extreme weather events such as polar vortexes that can reduce the EAB’s survival (48, 54).

In this study, we present a spatialized model for EAB that integrates predictions of phenology and climatic suitability to provide insight into the climatic risk of EAB being present and its seasonal development where climates are suitable for establishment. The model was developed for use in the DDRP (Degree-Days, Risk, and Pest event maps) platform (55, 56), which is an open-source decision support tool (https://github.com/bbarker505/ddrp_v2.git) to help detect, monitor, and manage invasive threats. DDRP has been operationalized at USPest.org (https://uspest.org/CAPS) to provide regularly updated (every 2−3 days) forecasts of phenology and climatic suitability for 16 invasive insect species in the conterminous United States (CONUS). The DDRP model for EAB improves upon previously developed models for this pest by integrating predictions of phenology and climate-based establishment risk, predicting phenology for all major life stages, incorporating within-population variation in phenology, and using a process-based approach to model climatic suitability based on thermal stress accumulation.

We then evaluated the performance of the model using available monitoring datasets and presence records for EAB. To date, assessing performance of DDRP models has been difficult because most modeled species are not currently established in CONUS (55, 57), and daily climate data sets needed for modeling are typically unavailable for regions where they are established (e.g., the native range). We capitalized on numerous phenological observations and presence records for EAB, as well daily climatic datasets from three different regions (China, Europe, and North America), to evaluate model performance for this species. Finally, we demonstrate the model by producing phenological event maps for North America and Europe for a particularly warm recent year, and by testing for temporal trends in EAB’s phenology and potential distribution over a recent 20-year period. Understanding the potential impacts of climate warming on EAB’s development and survival is important for planning surveillance and management programs for this pest (33, 58).

## 2 Methods

All analyses and modeling were conducted using R versions 3.6 or 4.1 (59, 60). Raster data processing and calculations were performed using the *raster* R package v. 3.5 (61) and *terra* package R package v. 1.5 (62). All plots were produced using the *ggplot2* R package 3.4.0 (63).

### 2.1 Overview of DDRP

**Figure 1:**
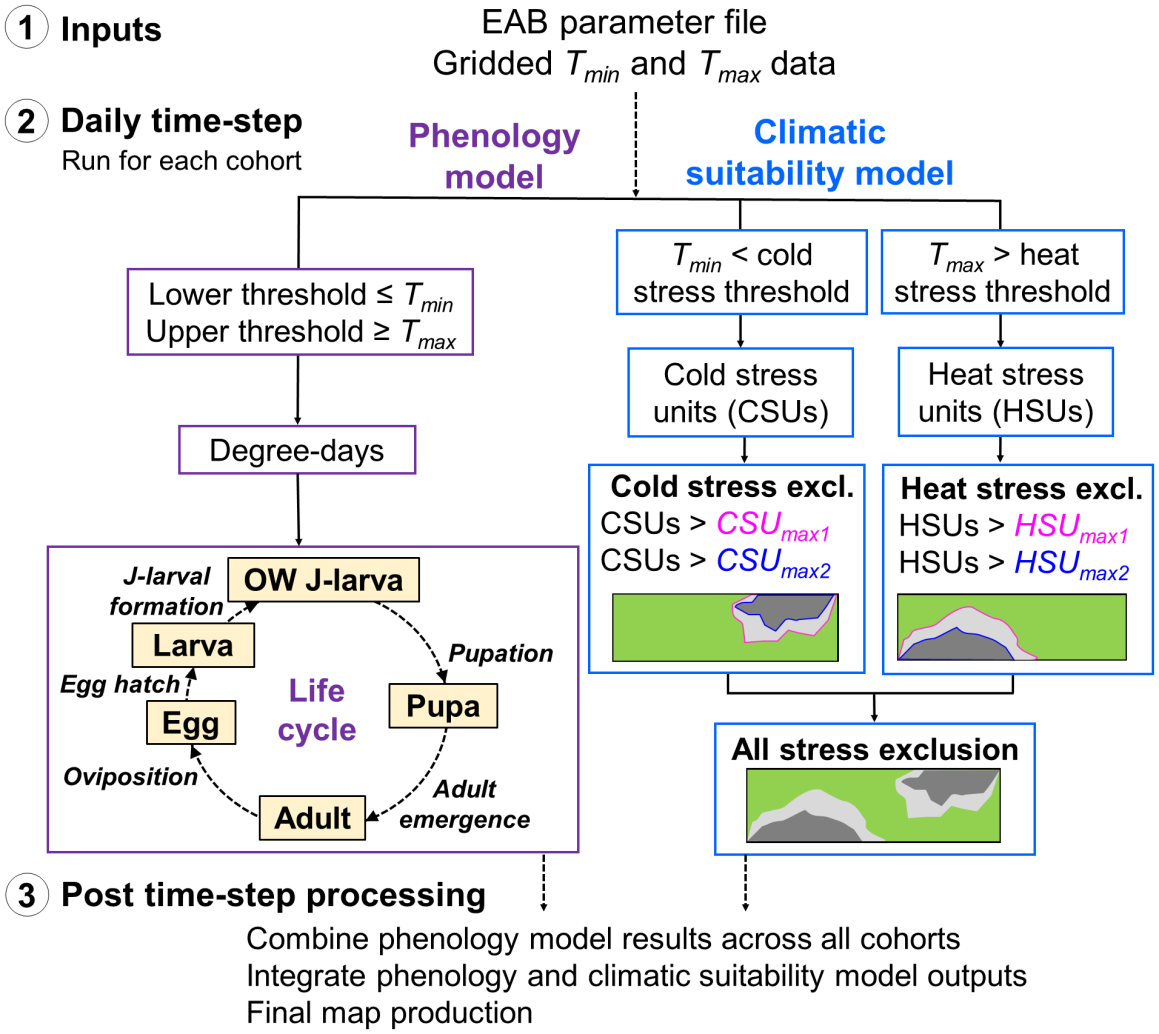
Schematic of the DDRP model framework for emerald ash borer (EAB). (1) Input data sets include the model parameter file and daily minimum and maximum temperature data (*T_min_* and *T_max_*, respectively). (2) Hollow boxes indicate calculations conducted at each daily time-step in the phenology and climatic suitability model (purple and blue boxes, respectively). The phenology model includes one phenological event (italic font) each for five life stages: the overwintered (OW) J-larva, pupa, adult, egg, and larva (instar 1 to J-larval formation). (3) After the daily time-step completes, DDRP combines phenology model results across all cohorts, integrates outputs of the phenology and climatic suitability models, and exports the final outputs as multi-layer raster (GeoTIFF) and summary map (PNG) files.

The mechanics of DDRP are detailed in Barker et al. (55). Briefly, DDRP uses a process-based modeling approach in which degree-days and climate stress are calculated daily and accumulate over time to model phenology and climatic suitability, respectively (**Figure 1**). Required inputs include gridded daily minimum and maximum temperature data (*T_min_* and *T_max_*, respectively) for a time frame of interest and a species parameter file (summarized in **Table 1**). Models include four separate life stages (egg, larva, pupa, and adult) plus a separately parameterized overwintering stage.

Phenology models often incorporate variation in phenology using a user-defined number of time-distributed cohorts that reflect the distribution of times in which the overwintering stage completes development. Thus, the daily accumulation of degree-days and accompanying life cycle transitions are carried out in separate bins starting from the completion of development of the overwintering stage of each cohort. For computational simplicity, individuals within each cohort are assumed to develop in synchrony. We typically apply seven cohorts because they approximate a normal distribution of development completion times and allow for reasonable model run times since the phenology model is run separately for each cohort.

DDRP uses accumulated stress unit totals for the year to delimit two levels of exclusion (i.e., moderate vs. severe stress exclusion) at any given location (**Figure 1**) (55). Areas not excluded by either moderate or severe temperature stress are part of the potential distribution and therefore at highest risk of establishment. Moderate stress may inhibit long-term establishment, with a high probability that short-term establishment could occur during favorable years, whereas areas under severe stress would likely prevent even short-term (one complete year) establishment.

Over the daily time-step, DDRP produces outputs that include maps of the potential distribution, number of generations, life stages present, and dates of phenological events. Additionally, it integrates maps produced by the phenology and climatic suitability model to provide insight into phenology (i.e., life stage present or the date of specific event) only in areas at risk of establishment.

### 2.2 Phenology model

Parameter values used in the final phenology model are provided in **Table 1**.

#### 2.2.1 Life cycle and overwintering stage

The model uses a start date of 1 January and assumes a one-year (univoltine) life cycle in which the J-larva is the overwintering stage (**Figure 1**). EAB develops through four larval instars before transitioning into a J-shape (mature 4^th^ instar) and overwintering via an obligatory diapause (or winter dormancy) phase, which is required for pupation and adult emergence to occur when the growing season begins (64). EAB is known to have a two-year (semivoltine) life cycle in cooler climates or when population densities are low (65–68), in which insects do not advance to the J-larval stage in a single growing season and require another year to complete development. In semivoltine populations, EAB overwinters in various larval instar stages, resulting in overlapping life stages (67–71). Phenology model predictions for semivoltine populations are relevant only to individuals in their second year of development (i.e., insects that overwintered as J-larvae).

#### 2.2.2 Thresholds, stage durations, and phenological events

We assigned all life stages of EAB a lower developmental threshold of 12.2°C (54°F) based on a re-analysis of development times reported by previous studies for the egg stage, egg to J-larvae stage, and oviposition longevity (**Supplementary Data 1**; **Supplementary Figures 1−3**) (32, 37, 64, 72). DDRP allows for different temperature thresholds for each stage, but there was insufficient data for deriving thresholds for certain stages or using a different threshold resulted in very similar predictions. For example, a lower threshold of 10°C (50°F) is commonly used to predict EAB adult phenology (5, 36, 73, 74), but applying this threshold to J-larvae and pupae did not improve predictive accuracy of first adult emergence (**Supplementary Data 2**). Additionally, the site-based phenology modeling tool that we use at USPest.org (https://uspest.org/risk/models) requires common thresholds, and these are presented as whole numbers in Fahrenheit scale for easy interpretation by end-users. Building the model for both platforms keeps models simpler and facilitates cross-comparison.

Degree-day requirements for the adult, egg, and larval (instar 1 to J-larval formation) stages were derived using the regression equations (**Supplementary Figures 1−3**) whereas those for the pupal stage were derived from previously reported values (37, 75). An upper developmental threshold of 36°C (97°F) for all life stages is based on studies showing that temperatures of *ca.* 35°C led to lower fecundity and survival rates in adults (32, 37, 76) and longer development times in larvae (32, 72). The model applies the single triangle calculation method with upper threshold (77) to calculate degree-days. It predicts five phenological events: pupation, adult emergence, oviposition, egg hatch, and J-larval formation (**Figure 1**).

**Figure 2:**
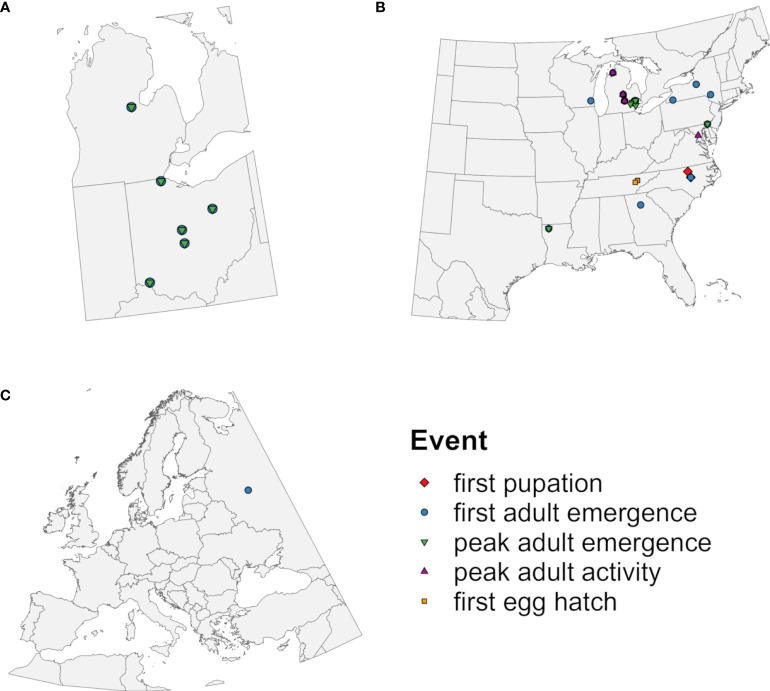
Geographic origins of observations of emerald ash borer used to **(A)** calibrate cohort parameters in the DDRP model and to **(B, C)** evaluate predictive accuracy of five phenological events. Symbols represent phenological events predicted by the model that were compared to observed events.

The phenology model applied time-distributed cohorts because within-site variation in the timing of emergence of EAB adults is well documented (36, 37, 67, 74). A monitoring dataset for EAB emergence at five locations (36) (**Figure 2A**) was used to calibrate model parameters that describe the completion of J-larval development (**Table 1**) because it provided insight into the full range and shape of the distribution of emergence times. As described in **Supplementary Data 3**, this involved calculating degree-day accumulation for emergence events using a lower developmental threshold of 12.2°C (**Supplementary Table S1**), visualizing and summarizing resulting values to create a matrix of possible parameter values (**Supplementary Figure S4**; **Supplementary Tables S2, S3**), and identifying the combination of parameter values that resulted in the lowest mean absolute error (MAE) between predicted and observed days of the year (DOYs) of first and 50% adult emergence (hereafter “peak” adult emergence). The application of seven cohorts combined with the final cohort parameter values resulted in distribution of adult emergence times that corresponded well with monitoring data (**Supplementary Figure S4**).

**Table 1:** Parameters used in the DDRP model for emerald ash borer.

Parameter	Code	Value
Lower developmental thresholds (°C)		
Egg	eggLDT	12.2
Larvae	larvaeLDT	12.2
Pupae	pupaeLDT	12.2
Adult	adultLDT	12.2
Upper developmental thresholds (°C)		
Egg	eggUDT	36.0
Larvae	larvaeUDT	36.0
Pupae	pupaeUDT	36.0
Adult	adultUDT	36.0
Stage durations (°C degree-days)		
Egg	eggDD	172
Larvae (instar 1 to J-larval formation)	larvaeDD	700
Pupae	pupDD	135
Adult	adultDD	145
Phenological events (°C degree-days)		
Appearance of pupae	OWEventDD	varies
Egg hatch	eggEventDD	172
Appearance of J-larvae	larvaeEventDD	700
Adult emergence (pupal development complete)	pupaeEventDD	135
Egg laying	adultEventDD	72
Cold stress		
Cold stress temperature threshold (°C)	coldstress_threshold	−31
Moderate cold degree-day (°C) limit	coldstress_units_max1	80
Severe cold degree-day (°C) limit	coldstress_units_max2	160
Heat stress		
Heat stress temperature threshold (°C)	heatstress_threshold	38
Moderate heat stress degree-day (°C) limit	heatstress_units_max1	75
Severe heat stress degree-day (°C) limit	heatstress_units_max2	150
Cohorts		
Degree-days (°C) to complete J-larval development (average)	distro_mean	200
Degree-days (°C) to complete J-larval development (variation)	distro_var	15000
Minimum degree-days (°C) to complete J-larval development	xdist1	60
Maximum degree-days (°C) to complete J-larval development	xdist2	350
Shape of the distribution	distro_shape	lognormal
Other		
Order of stages	stgorder	OL, P, A, E, L
Obligate diapause (1 = TRUE)	obligate_diapause	1
Degree-day calculation method	calctype	triangle

OL, Overwintered larvae (J-larvae); P, pupae; A, adult; E, egg; L, larvae.

Differences between predicted and observed DOYs of first and peak adult emergence were visualized with scatterplots (with smooth lines) and summarized with estimates of their range and bias. Bias was estimated as the average amount by which predicted DOYs are greater than observed DOYs, in which negative values would indicate model underprediction (too early) whereas positive values would indicate overprediction (too late).

#### 2.2.3 Phenology model validation

We used field-collected phenological observations to evaluate the accuracy of predicted DOYs for phenological events in EAB. Observations of first pupation (78, 79), first adult emergence, peak adult emergence, peak adult activity (32, 34, 37, 67, 71, 74, 79–83), and first egg hatch (84) used for model validation were derived from the literature, the *Nature’s Notebook* database [accessed 10 Nov 2022 using the *rnpn* R package version 1.25 (85, 86)], and monitoring datasets collected for this study (T. Petrice and J. Duan; **Supplementary Table S4**). This resulted in 60 observations collected from 23 locations in nine states in the eastern United States between 2003 and 2022 (**Figure 2B**) and from one location (Moscow) in European Russia in 2013 and 2014 (**Figure 2C**; **Table 2**). Only 41 observations were independent in terms of location and year because the monitoring study reported multiple phenological events over the year (**Supplementary Table S5**). We did not include observations that were likely derived from semivoltine individuals in their second year of development (67, 71).

**Table 2:** Summary of field observations used to validate phenological event predictions produced by the DDRP model for emerald ash borer.

Observed event	N_loc_	N_obs_	Observation years	Phenological event
first pupation	2	3	2017, 2020, 2021	pupal development (earliest)
first adult emergence	16	30	2003, 2004, 2010, 2013, 2014, 2016−2022	adult emergence (earliest)
peak adult emergence	7	8	2003−2005, 2016, 2020	adult emergence (average)
peak adult activity	6	16	2006, 2007, 2016−2019	egg-laying (earliest)
egg hatch	2	3	2016, 2017	egg hatch (earliest)

Each observed event has a corresponding predicted event in the model [earliest, earliest day of year (DOY) across cohorts; average, weighted average of DOYs across cohorts]. N_loc_, number of locations; N_obs_, number of observations.

Models for the eastern United States used daily estimates of *T_min_* and *T_max_* from the PRISM database at a spatial resolution of 4 km^2^ (https://prism.oregonstate.edu) (87) whereas those for Europe used estimates from the E-OBS database at a spatial resolution of 0.1° (*ca.* 11.1 km^2^; https://surfobs.climate.copernicus.eu, accessed 19 April 2022) (88). DOYs for first pupation, first adult emergence, and first egg hatch were calculated as the earliest DOY for pupal development, adult emergence, and egg hatch across cohorts, respectively. DOYs for peak adult activity were calculated as the weighted average of DOYs for oviposition across cohorts. Predictions were extracted from corresponding grid cells in the phenological map outputs produced by DDRP. Five observations from Michigan lacked precise coordinate data (**Supplementary Table S4**) so predictions were obtained by averaging predictions within their city of origin (Ann Arbor, Detroit, Novi, and Troy). A shapefile of city boundaries was obtained from the U.S. Census Bureau's Master Address File / Topologically Integrated Geographic Encoding and Referencing (TIGER) database.

For each phenological event, we visualized differences between predicted and observed DOYs with scatterplots and calculated descriptive statistics (MAE and bias). Additionally, we performed a two one-sided (TOST) test for equivalence using the *equivalence* R package 0.7.2 (89, 90) to evaluate whether predicted and observed DOYs for adult events were equivalent. Tests did not assume equal variances between groups. Statistical equivalence can be demonstrated if the two one-sided 95% confidence intervals (CIs) are completely contained within a specified equivalence interval for the difference in means (δ) (i.e., bias). For each event, we conducted a test that applied an equivalence interval of seven days (−7 < δ < 7) and another that applied an equivalence interval of 14 days (−14 < δ < 14). A narrower equivalence interval makes it more difficult to reject the null hypothesis of differences in means. Equivalence tests were not performed for first pupation and first egg hatch owing to very small sample sizes.

### 2.3 Climatic suitability model

We developed and validated a climatic suitability model for EAB using a combination of eco-physiological information and georeferenced presence records from China (N = 148), European Russia and Ukraine (hereafter Europe; N = 139), and North America (N = 2947). Presence records were derived from peer-reviewed literature, theses, reports, the NAPIS database (https://cmr.earthdata.nasa.gov/search/concepts/C1214608223-SCIOPS, accessed 29 Aug 2022), the Global Biodiversity Information Facility (91) (http://gbif.org, accessed 27 Dec 2022), and CERIS Pest Tracker (https://pest.ceris.purdue.edu, accessed 27 Dec 2022). Of the 3234 presence records, 1068 were spatially resolved only to the level of a county or city, which is typically a coarser scale than climate data. However, predictive uncertainty was not an issue for these locations because none occurred near the predicted range limits for EAB (see ‘3.2 Results’). Presence records were deposited in Zenodo (https://doi.org/10.5281/zenodo.7493142).

Climatic suitability models used daily estimates of *T_min_* and *T_max_* for the most recent 20-year time frame available for each focal region. These included (1) the CDAT dataset for China for 1999 to 2018 at a spatial resolution of 0.1° (92)(accessed on 28 Jun 2022; https://zenodo.org/record/5513811#.YtnLNXbMIuU), (2) the Daymet dataset for North America for 2002 to 2021 at a spatial resolution of 1 km^2^ (93, 94) (https://daymet.ornl.gov/getdata, accessed 13 Jul 2022), and (3) the E-OBS dataset for Europe for 1999 to 2018. E-OBS data for 2019 and 2020 were excluded because of a large amount of missing data in European Russia. Daymet data were chosen over PRISM data because they include climate estimates for Canada; however, they were cropped to a maximum latitude of 60°N and aggregated to a spatial resolution of 4 km^2^ to decrease model run times.

#### 2.3.1 Climatic suitability model parameter calibration

Parameter values used in the final climatic suitability model are provided in **Table 1**.

##### 2.3.1.1 Cold stress parameters

To identify an appropriate cold stress threshold, we extracted estimates of *T_min_* of the coldest week for each presence record from the provinces of Inner Mongolia, Heilongjiang, and Jilin in northeastern China. These provinces include some of the coldest areas of the species’ known distribution (95). Daily CDAT data (1999 to 2018) were averaged and then aggregated to a weekly resolution to better reflect longer term (i.e., multi-day) cold stress experienced by EAB, which is capable of surviving through extended periods of sub-zero temperatures (54, 96). According to this analysis, all records occurred in areas with weekly *T_min _* values ≥ −31°C (**Supplementary Figure S5**). This finding is consistent with work showing that EAB is killed by internal ice formation that occurs at temperatures around −31°C (97), and with laboratory studies of EAB’s cold hardiness (96, 98).

The average number of consecutive days during winter months (December, January, and February) that daily average *T_min _* fell below −31°C was calculated to help define cold stress limits. The severe cold stress limit in DDRP was set to correspond roughly with areas that experienced more than 10 consecutive days below −31°C (**Supplementary Figure S5**). Mortality rates of cold-acclimated EAB larvae were *ca.* 20 to 60% after exposure to temperatures between −30°C and −35°C for one week (96), which suggests that exposure of EAB to these temperatures for a week may not prevent long-term establishment. One field study reported severe mortality (93%) in populations that experienced temperatures below −30°C for a three-day period; however, deacclimation of larvae resulting from unusually warm winter conditions likely explained low survival rates (54).

We delineated the moderate cold stress limit to correspond roughly with areas that experienced a daily average *T_min_* < −31°C for five to 10 consecutive days during winter months (**Supplementary Figure S5**). The moderate cold stress limit was further calibrated by minimizing the number of records that were excluded in a model for 2001, which was an extreme year in terms of cold stress accumulation. Several records were excluded by severe (N = 1) or moderate (N = 16) cold stress in 2001 even after calibrations. However, we opted to retain parameter values because most of these records (N = 16) were not excluded for other years.

##### 2.3.1.2 Heat stress parameters

EAB may survive at temperatures near 56°C for short periods of time (≤ 60 min) (99, 100), but the impact of less extreme heat levels on survival are not well understood. Following the same approach used for cold stress parameter calibration, we averaged daily Daymet data (2001 to 2021) and extracted data on weekly and daily estimates of *T_max_* data for presence records from the southern United States, which is the hottest area for which records existed. According to this analysis, all records had average weekly *T_max_* ≤ 38°C and six occurred in areas of northcentral Texas that experienced daily average *T_max_* > 38°C for 2−4 days (**Supplementary Figure S6**).

We set the severe and moderate heat stress limits to correspond roughly with areas that experienced average daily *T_max_* for 40 and 90 consecutive days during summer months (as delineated in **Supplementary Figure S6**), respectively, to avoid underpredicting the pest’s potential distribution in hot areas. The moderate heat stress limit was further calibrated by minimizing the number of records that were excluded in the model that used data for an extreme year (2011) in terms of heat stress accumulation (**Supplementary Figure S6**).

#### 2.3.2 Climatic suitability model validation

We assessed whether DDRP correctly included presence records not used for model calibration in the potential distribution for each of 20 modeled years. This analysis included records from Europe (N = 139), China (N = 107, not including records from Heilongjiang, Inner Mongolia, and Jilin provinces), and North America (N = 2790, not including records from the southern United States). Maps were combined to identify areas that were consistently included in the potential distribution across 20 years. Combined maps were compared to digitized maps of native *Fraxinus* species for North America (101) and Europe (102) to assess potential range limitations due to host plant availability. We were unable to find digitized maps of native *Fraxinus* species for China.

### 2.4 Demonstration

We produced phenological event maps of first adult emergence and first egg hatch for EAB in 2021 for North America and Europe to provide insight into its phenology and potential distribution during a particularly warm year. The summer of 2021 was tied with 1936 for the warmest on record in CONUS (NOAA website https://www.ncei.noaa.gov/news/national-climate-202108, last accessed 22 Jan 2023) and was the hottest on record for Europe (European State of the Climate 2021, https://climate.copernicus.eu/esotc/2021, last accessed 22 Jan 2023). The model used Daymet data for North America and E-OBS data for Europe.

We tested for trends in the DOY of first adult emergence and climate stress exclusions over a 20-year period to explore potential changes in EAB’s phenology and potential distribution due to climate warming. Tests used the ‘Mann-Kendall’ function in the *Kendall* R package v. 2.2.1 (103) and excluded grid cells with missing data (Europe only). The Mann-Kendall statistical test (104, 105) is a rank-based non-parametric method that is widely used for time series of remote sensing data (106, 107). Analyses of climate stress exclusions were only conducted for North America because Europe was climatically suitable for most years (see ‘3.2 Results’). We used absolute values for climate stress exclusion values in DDRP (0 = no stress exclusion, −1 = moderate stress exclusion, and −2 = severe stress exclusion) to make trends easier to interpret (e.g., a decreasing trend would indicate decreasing climate stress over time). Trends were considered statistically significant at the P = 0.1 level because Mann-Kendall tests may have low power for time-series data (107).

## 3 Results

### 3.1 Phenology model

**Figure 3:**
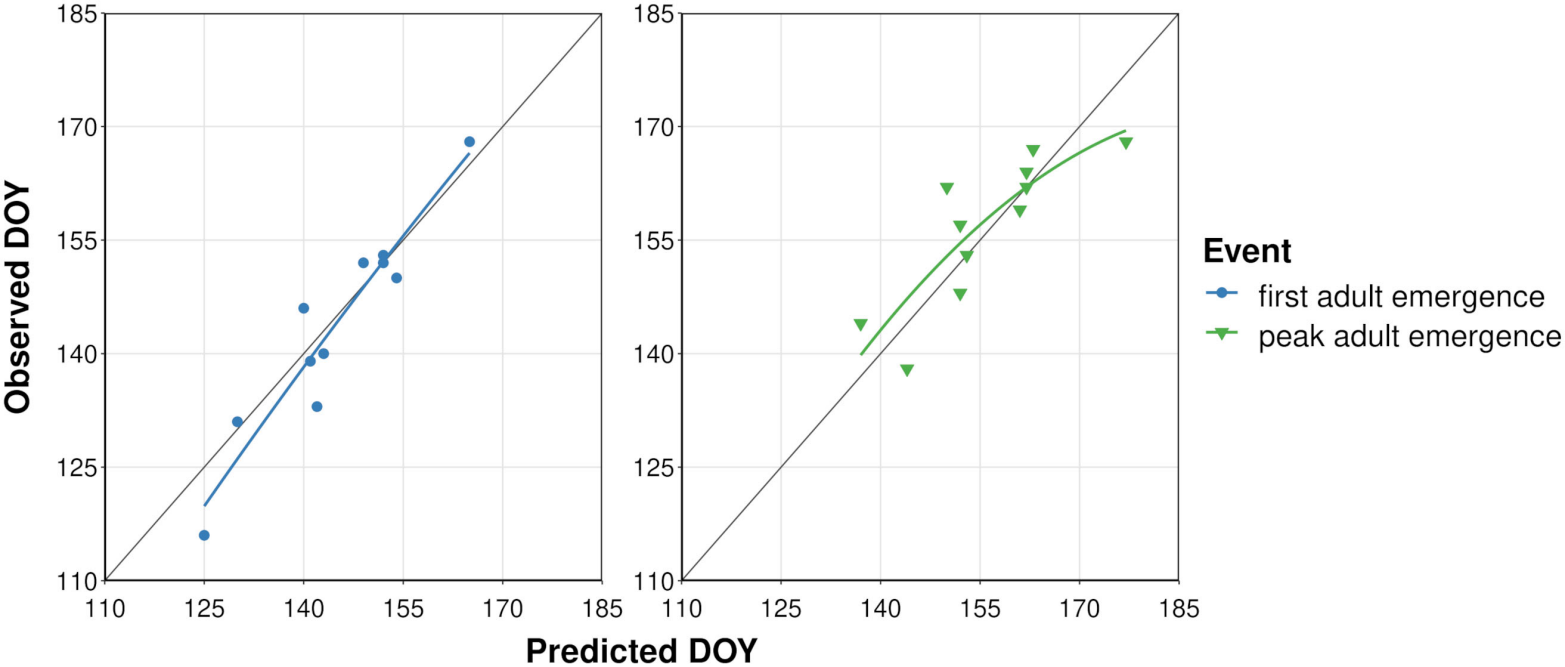
Scatterplots showing predicted vs. observed days of year (DOYs) of first and peak adult emergence of emerald ash borer based on the dataset used to calibrate the phenology model. Solid colored lines represent the curved line of best fit calculated with linear regression. Dark gray line represents a 1:1 relationship between predicted and observed DOYs.

The process of filtering combinations of five cohort parameter values to determine overwintering cohort settings resulted in 22 parameter combinations (**Supplementary Table S3**). The lowest combined MAE for first and peak adult emergence (i.e., sum of MAE for both events) was obtained for the model that used a low bound = 60, mean = 200, high bound = 350, and variance = 15000 of development completion times [in degree-days (°C), DDC] for J-larvae. Accordingly, adult emergence was predicted to occur between 217 and 460 DDCs (**Supplementary Table S6** and **Figure S4**). Predicted DOYs exhibited a linear relationship with observed DOYs (**Figure 3**), although the line of fit for peak adult emergence curved downward at higher DOYs. The MAE between predicted and observed DOYs for first and peak adult emergence were 3.7 and 4.6 days, respectively (**Table 3**). On average, first adult emergence was overpredicted by 1.2 days [standard deviation (SD) = 4.8] whereas peak adult emergence was underpredicted by 0.8 days (SD = 6.1).

**Table 3:** Summary statistics for five phenological events predicted by the phenology model for emerald ash borer based on datasets used for model calibration and validation.

Analysis	Event	N_obs_	MAE	Bias	SD	Range
calibration	first adult emergence	11	3.7	1.2	4.8	−6 to 9
calibration	peak adult emergence	11	4.6	−0.8	6.1	−12 to 9
validation	first pupation	3	12.0	8.7	15.8	−5 to 26
validation	first adult emergence	30	7.4	1.6	9.2	−19 to 23
validation	peak adult emergence	8	8.4	−0.1	10.2	−14 to 14
validation	peak adult activity	16	7.2	−0.9	8.2	−12 to 14
validation	first egg hatch	3	13.7	−7.7	16.0	−23 to 9

The number of observations (N_obs_), mean absolute error (MAE), bias with standard deviation (SD), and range of differences between predicted and observed dates (in days) are shown for each event. MAE, the average absolute difference of (observed – predicted); Bias, average of (predicted – observed); SD, standard deviation of (predicted – observed).

**Figure 4:**
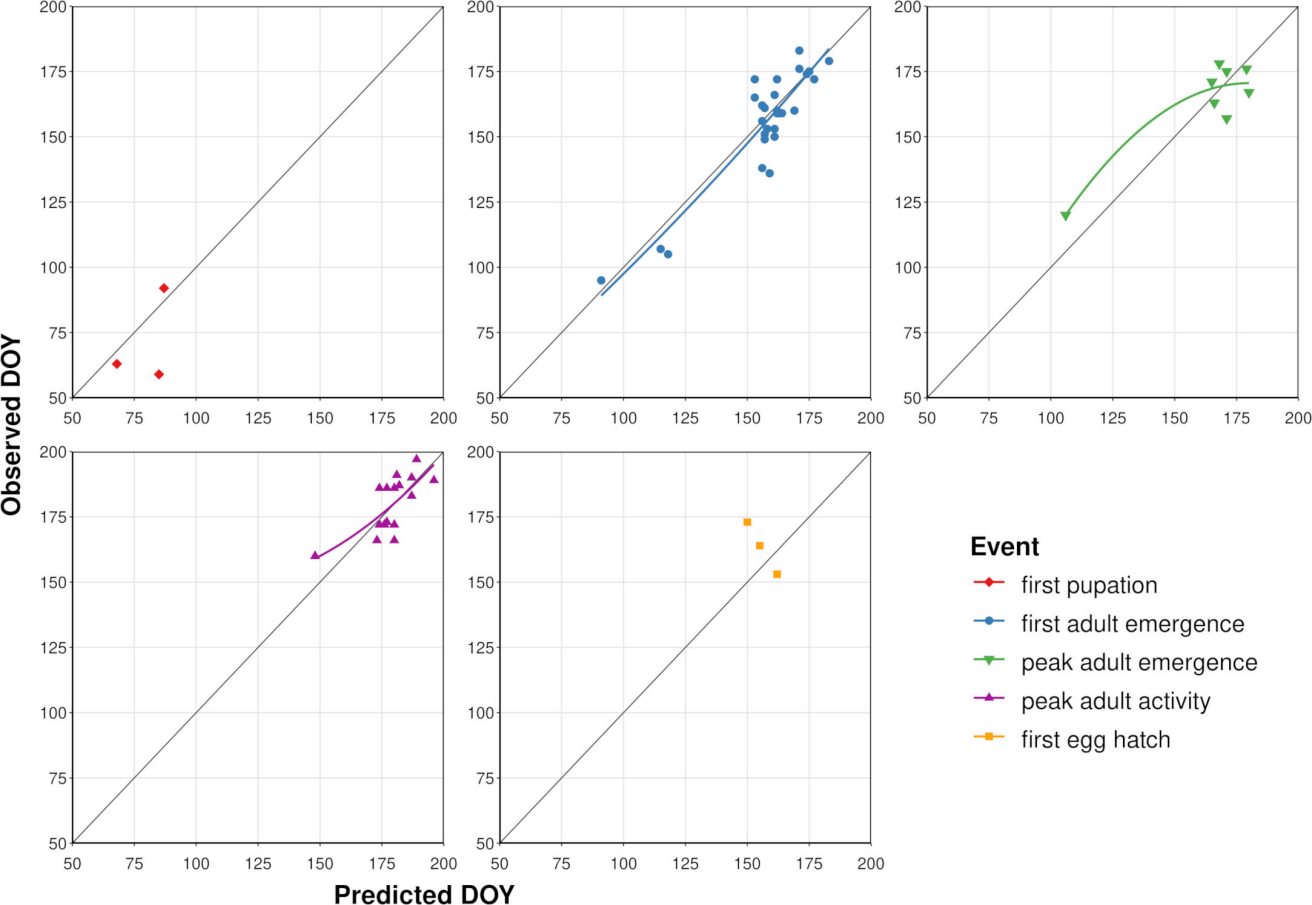
Scatterplots showing predicted vs. observed days of year (DOYs) for five phenological events in emerald ash borer based on the dataset used to validate the phenology model. Solid colored lines represent the curved line of best fit calculated with linear regression (not shown for first pupation and egg hatch owing to small sample sizes). Dark gray line represents a 1:1 relationship between predicted and observed DOYs.

According to model validation analyses, predicted DOYs for phenological events in adults exhibited mostly linear relationships with observed DOYs (**Table 3**; **Figure 4**). However, a single observation from Washington DC (34) resulted in a slight upward curve at lower DOYs for peak adult activity, whereas a single observation from Louisiana (80) resulted in a downward curve for peak adult emergence at lower DOYs. MAE values for first adult emergence, peak adult emergence, and peak adult activity were 7.4, 8.4, and 7.2 days, respectively. On average, first adult emergence was overpredicted by 1.6 days (SD = 9.2) whereas peak adult emergence and peak adult activity were underpredicted by 0.1 (SD = 10.2) and 0.9 days (SD = 8.2), respectively. Predicted DOYs for first pupation and first egg hatch exhibited lower correspondence with observed DOYs (**Figure 4**) and were less accurate (MAE = 12.0 and 13.7 days, respectively) than predictions of adult events. However, sample sizes for these two events were very small (N = 3 observations each).

**Figure 5:**
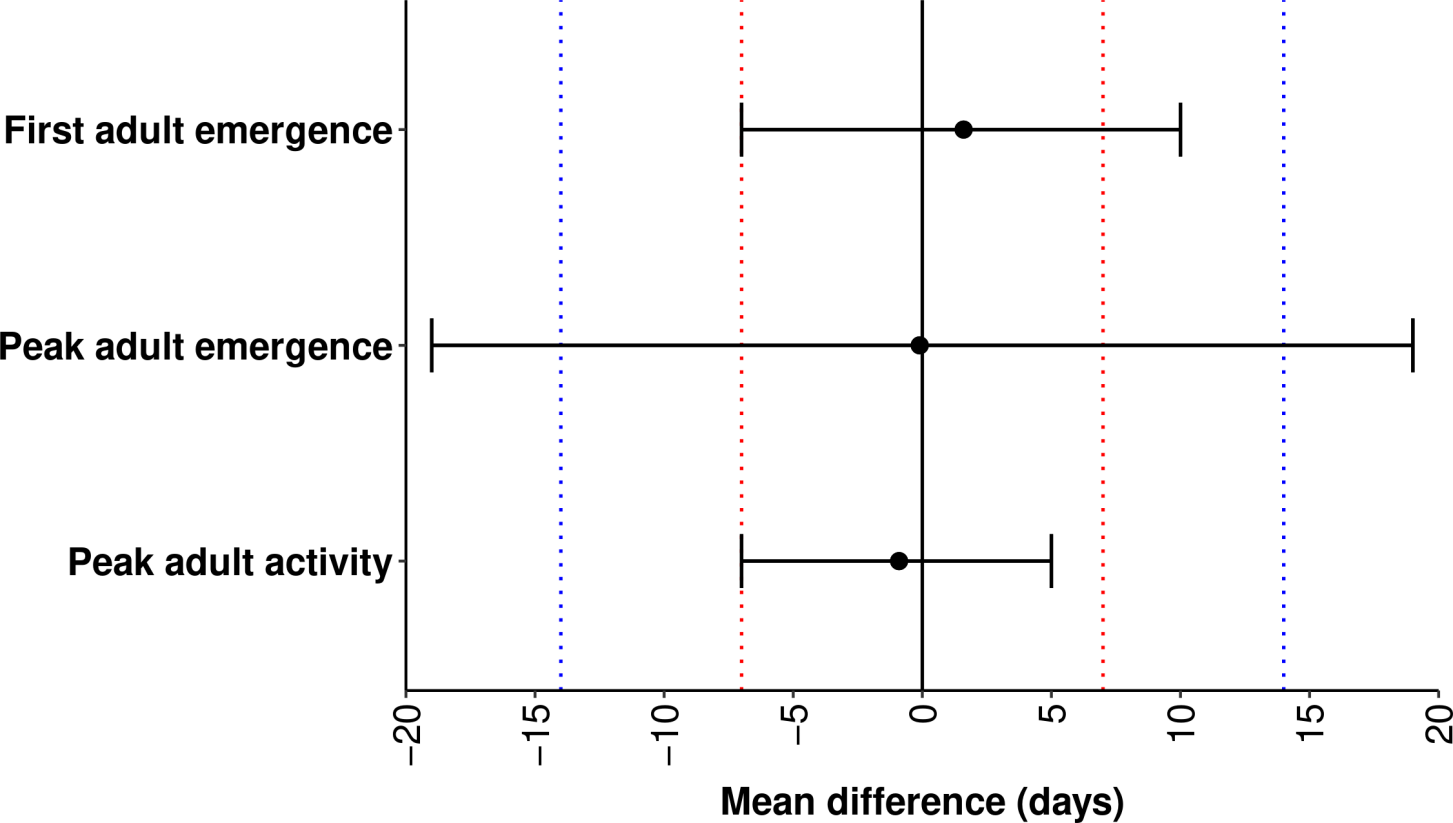
Results of equivalence tests for adult phenological events in emerald ash borer. The two one-sided 95% confidence intervals (black horizontal line) for differences between the means of predicted and observed days of year (solid circle) are shown for each event. Red and blue dotted lines depict the equivalence interval of seven days and 14 days, respectively.

According to equivalence tests for first adult emergence and peak adult activity, the two one-sided 95% CIs for difference between the means of predicted and observed DOYs were completely contained in the equivalence interval of 14 days (**Figure 5**), indicating that the means were statistically equivalent (no bias). However, the CIs for the difference between the means of predicted and observed DOYs for first adult emergence exceeded the upper equivalence bound of seven days, indicating overprediction of up to seven days. The CIs for the difference between the means of predicted and observed DOYs for peak adult emergence exceeded both the lower and upper bounds of the equivalence interval of 14 days, indicating under- and over-prediction of up to 14 days.

### 3.2 Climatic suitability model

**Figure 6:**
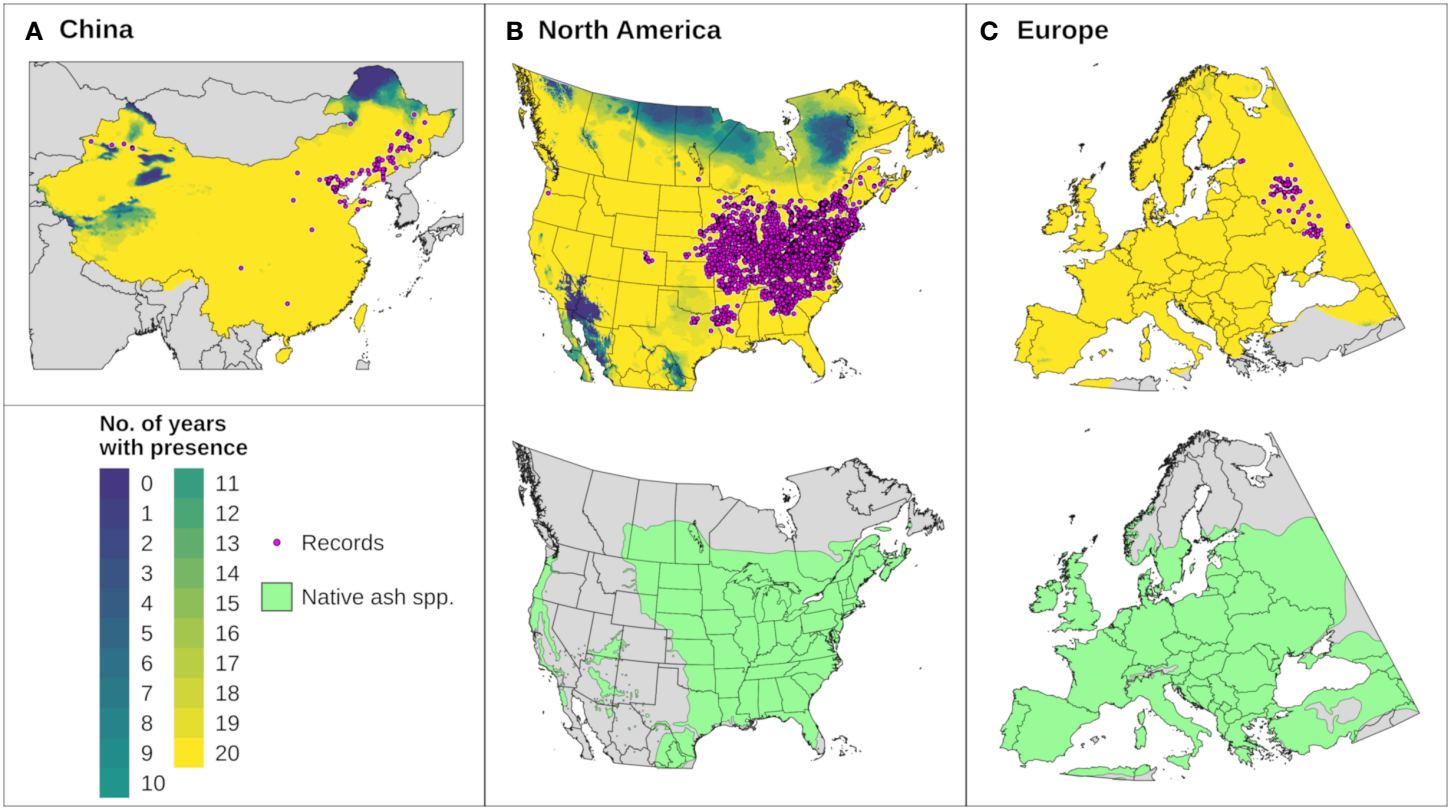
The modeled potential distribution for emerald ash borer (EAB) in **(A)** China, **(B)** North America, and **(C)** Europe according to DDRP runs for 20 recent years (top maps). Yellow areas were included in the potential distribution for all 20 years, areas with cooler colors were excluded by climate stress for one or more years, and gray areas lack predictions owing to missing climate data. Pink circles depict the approximate locations of presence records used for model calibration and validation. Bottom maps in **(B, C)** depict the range of native ash (*Fraxinus*) species (green shading) in North America (16 species) and Europe (three species) to provide insight into possible range restrictions in EAB due host plant availability. Spatial data for native ash species in China were unavailable.

**Figure 7:**
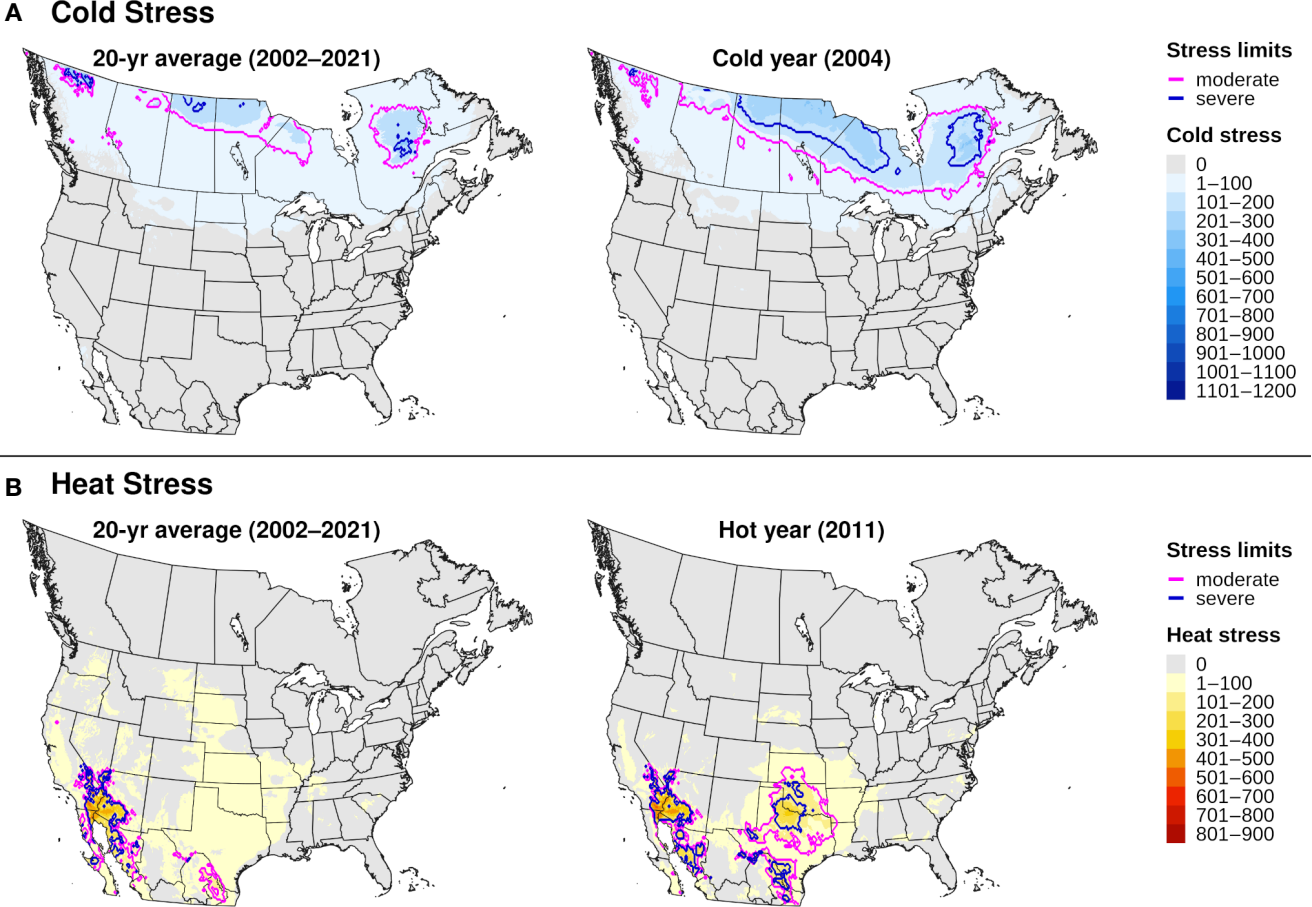
Maps of annual **(A)** cold stress and **(B)** heat stress accumulation for emerald ash borer in North America produced by DDRP. Results based on 20-year climate averages (left maps) are compared to those based on climate data for an extreme year (right maps) in terms of cold or heat stress accumulation (2004 and 2011, respectively).

**Figure 8:**
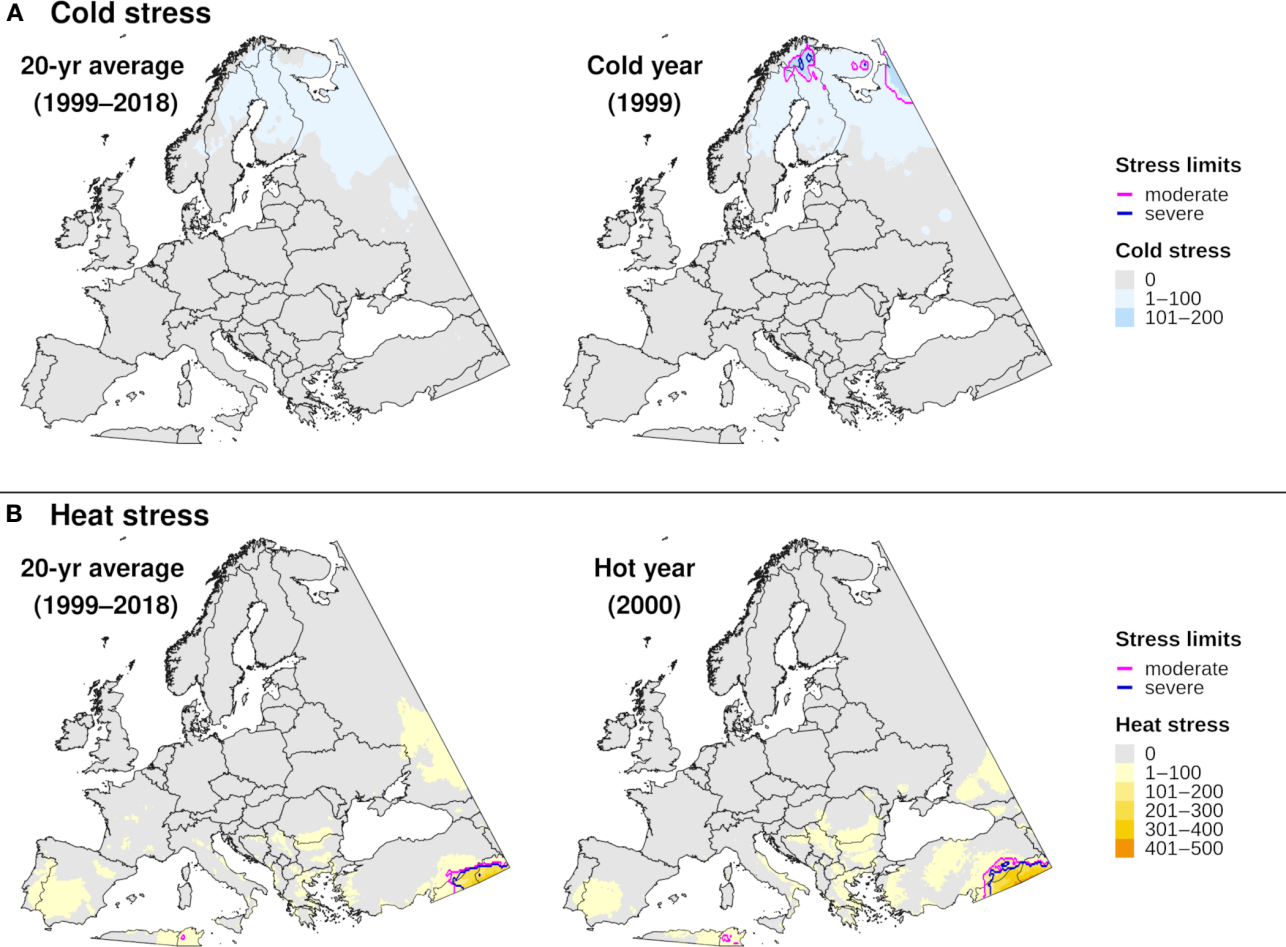
Maps of annual **(A)** cold stress and **(B)** heat stress accumulation for emerald ash borer in Europe produced by DDRP. Results based on 20-year climate averages (left maps) are compared to those based on climate data for an extreme year (right maps) in terms of cold or heat stress accumulation (1999 and 2000, respectively).

The climatic suitability model correctly predicted presence of EAB for 99.9% (3024/3028) of presence records used for model validation, which provides evidence for high model sensitivity. The potential distribution based on climate data for 20 years included most of China (**Figure 6A**), and it overlapped with the ranges of all native *Fraxinus* species in North America (**Figure 6B**) and Europe (**Figure 6C**). In North America, areas predicted to be excluded by cold stress for at least one modeled year corresponded roughly with the northern range edges (*ca.* north of 49 °N in continental areas) of green ash and black ash, *F. pennsylvanica* Marsh and *F. nigra* Marsh, respectively (**Figure 6B**). However, a few areas along these range edges were excluded by moderate cold stress during an extreme year in terms of cold stress accumulation (1999; **Figure 7A**). Conversely, cold stress accumulation in Europe did not exceed EAB’s limits in any region, except for some small areas of the northernmost latitudes of Europe and European Russia in 1999 (**Figure 8A**).

Heat stress played a minor role in shaping EAB’s potential distribution within the ranges of native *Fraxinus* species in North America and Europe (**Figures 7B**, **8B**). However, parts of the ranges of Mexican ash (*F. berlandieriana* DC) and littleleaf ash (*F. greggii* Gray) in northeastern Mexico as well as California ash (*F. dipetala* Hook. & Arn.) in Baja California were excluded by moderate heat stress (**Figure 7B**). Areas excluded by moderate and severe heat stresses expanded throughout this area and in southern United States (Texas and Oklahoma) during an extreme year in terms of heat stress accumulation (2011).

### 3.3 Demonstration

**Figure 9:**
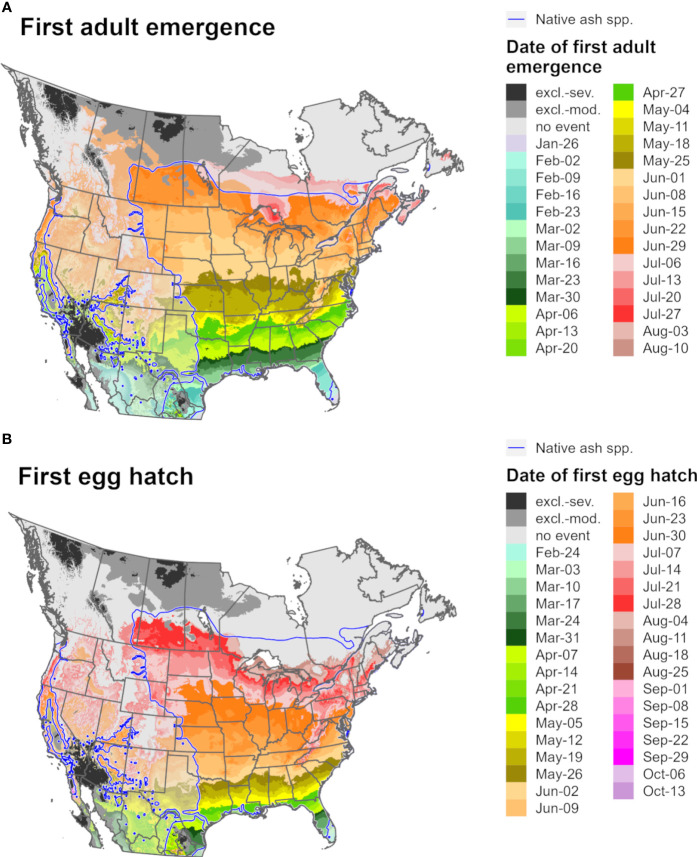
Maps of the predicted dates of **(A)** first adult emergence and **(B)** egg hatch for emerald ash borer for North America for 2021 produced by DDRP. Phenological predictions occurring outside of the range native ash (*Fraxinus*) species (blue line) are semi-transparent. Maps include estimates of climatic suitability, where long-term establishment is indicated by areas not under moderate (excl.-moderate, medium gray) or severe (excl.-severe, dark gray) climate stress exclusion. Areas with insufficient degree-day accumulation for an event to occur are indicated with light gray.

**Figure 10:**
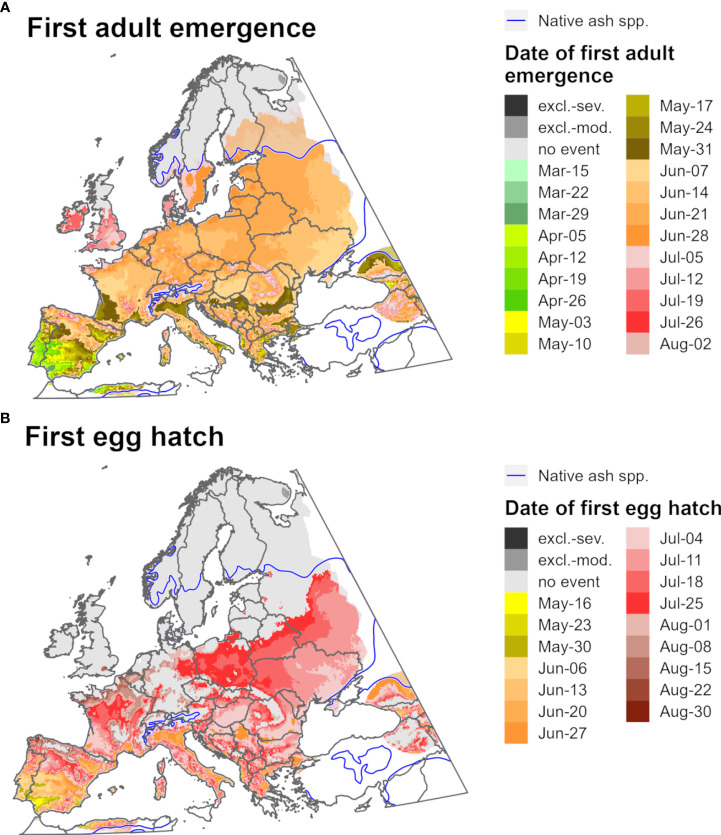
Maps of the predicted dates of **(A)** first adult emergence and **(B)** egg hatch for emerald ash borer for Europe for 2021 produced by DDRP. Phenological predictions occurring outside of the range native ash (*Fraxinus*) species (blue line) are semi-transparent. Maps include estimates of climatic suitability, where long-term establishment is indicated by areas not under moderate (excl.-moderate, medium gray) or severe (excl.-severe, dark gray) climate stress exclusion. Areas with insufficient degree-day accumulation for an event to occur and missing climate data are indicated with light gray and white, respectively.

Predictions of first adult emergence and egg hatch for EAB in 2021 varied substantially by latitude. For most of Europe and northern parts of North America, first adult emergence occurred between June and July (**Figures 9A**, **10A**) whereas egg hatch occurred between late June and August (**Figures 9B**, **10B**). The earliest dates for these events in North America were in southwestern areas (Mexico, southern Texas, and Florida) and latest in southeastern Canada, whereas in Europe the earliest dates were in Spain and latest in the United Kingdom and Scandinavia. There was insufficient degree-day accumulation for egg hatch to occur in parts of the ranges of *Fraxinus* species in southern Canada and northern Europe (e.g., in Ireland, Great Britain, and Scandinavia), which suggests that EAB may be unable to complete its life cycle in these areas. Within the host ranges, moderate cold stress was predicted for a small area of southern Saskatchewan and Manitoba in Canada.

**Figure 11:**
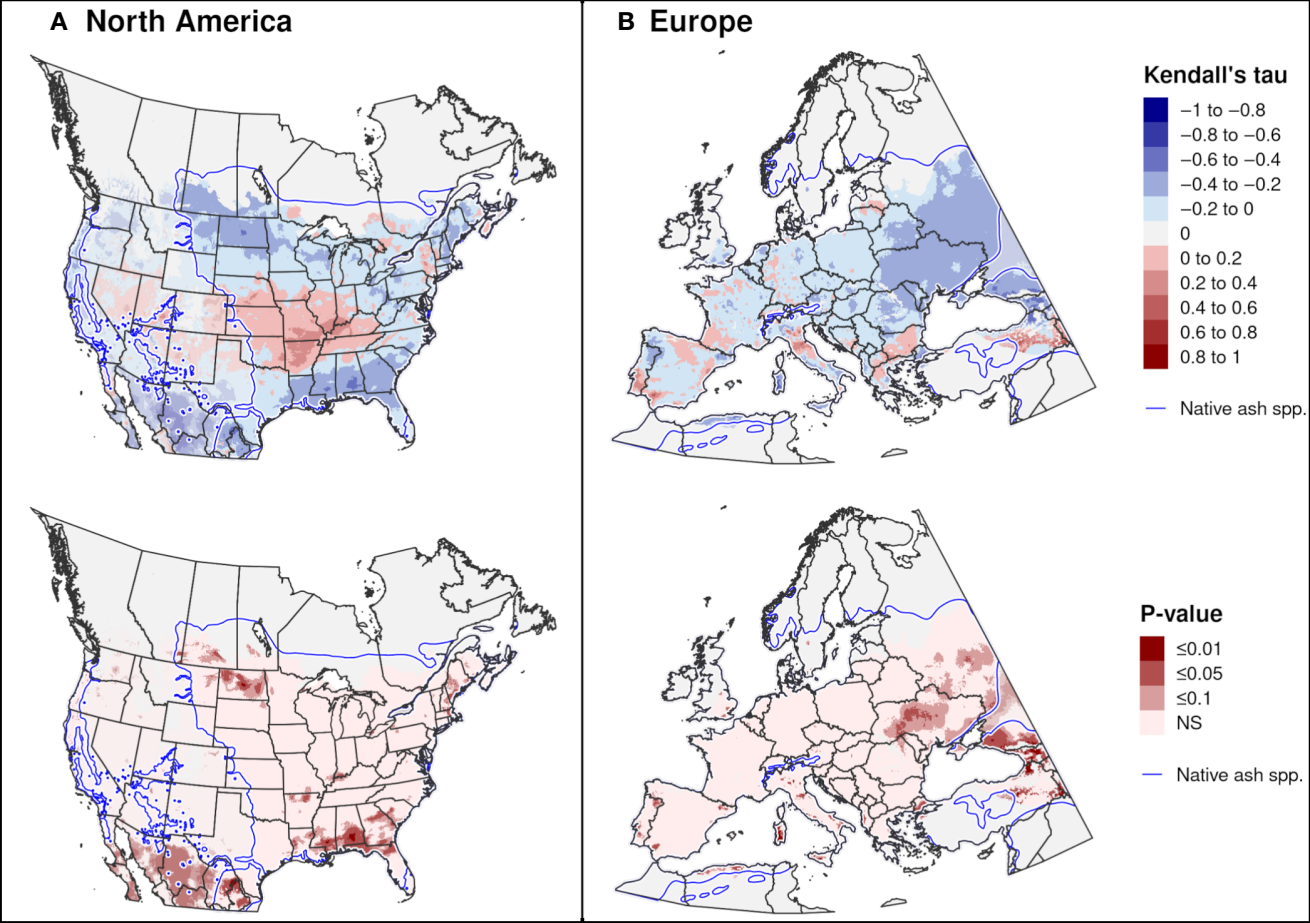
Trends in predicted dates of first adult emergence for emerald ash borer over a 20-year period in **(A)** North America (2002−2021) and **(B)** Europe (1999−2018). The Kendall tau metric varies from −1 and 1, where positive values indicate an increasing trend and negative values indicate a decreasing trend (top maps). Statistically significant trends are indicated (*P* ≤ 0.1; bottom maps). Predictions occurring outside of the range of native *Fraxinus* species (blue line) are semi-transparent. Areas with no trend, insufficient degree-day accumulation for emergence to occur, or missing climate data for any years are indicated with light gray.

Trends in the DOY of first adult emergence over 20 recent years were significant in only a few areas. Significant trends towards earlier emergence in North America occurred in both southern areas (parts of Mexico and the southeastern United States) and northern areas (parts of New England and the Midwest; **Figure 11A**), whereas in Europe they occurred predominantly in central-eastern regions (Ukraine and European Russia; **Figure 11B**). A significant trend towards later adult emergence were predicted in a few small areas of the midwestern United States and eastern Turkey.

**Figure 12:**
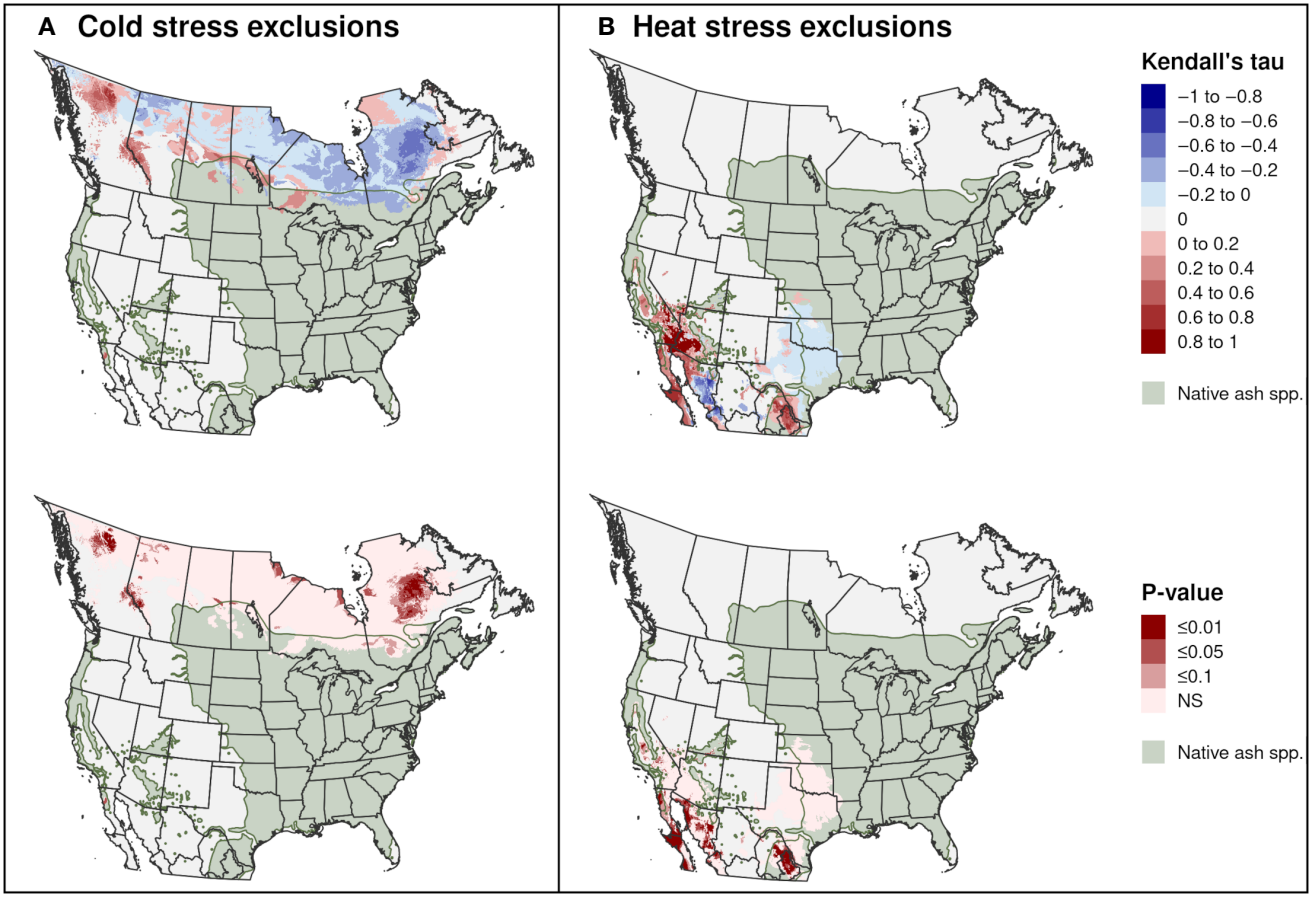
Trends in exclusions by **(A)** cold stress and **(B)** heat stress for emerald ash borer in North America between 2002 and 2021. The Kendall tau metric varies from −1 and 1, where positive values indicate an increasing trend and negative values indicate a decreasing trend (top maps). Statistically significant trends are indicated (*P* ≤ 0.1; bottom maps). The range of 16 native *Fraxinus* species is shown to provide insight into possible range restrictions in EAB due host plant availability. Areas with no trend are indicated with light gray.

Significant trends in range-limiting climate stress for EAB in North America between 2002 and 2021 occurred predominantly in areas outside of the range of native *Fraxinus* species. Areas excluded by cold stress declined in parts of Quebec, the Hudson Bay area in central Canada, and northern Alberta, whereas they increased in parts of western Canada (**Figure 12A**). Areas excluded by heat stress significantly increased in parts of northern Mexico and the southwestern United States, including in parts of the ranges of *F. berlandieriana* and *F. greggii* in northeastern Mexico and *F. dipetala* in Baja California (**Figure 12B**).

## 4 Discussion

### 4.1 Phenology model

According to model validation analyses, predicted DOYs for phenological events in adults exhibited strong concordance with observed DOYs, with mean absolute errors of *ca.* 7 days and low estimates of bias. However, an equivalence test indicated that the mean predicted DOY of first adult emergence was overpredicted by as much as seven days, which is concerning because surveillance and management tactics that target adults are often employed several days before adult emergence (5, 30). As a precautionary measure, activities such as installing detection devices and applying insecticide treatments could be implemented at least a week before the predicted date of first adult emergence to help avoid the potential consequences of model overprediction (e.g., failing to detect beetles that had already emerged).

Additional phenological observations collected from across a latitudinal range are needed to conduct a more robust evaluation of model performance. For example, high levels of under- and over-prediction of the DOY of peak adult emergence (±14 days) according to equivalence tests may be partly due to low statistical power owing to a small sample size (N = 8 observations). Sample sizes were particularly small for first pupation and first egg hatch (N = 3 observations each), so we refrained from interpreting results of analyses for these events. Additionally, observations of all life stages are needed from southern populations to better evaluate model performance in warmer climates. EAB’s activity and development in areas with warm climates are not well understood because the pest began spreading into the southern United States only over the past decade (14, 108, 109). To date, monitoring data collected from the southern United States indicate that EAB overwinters primarily as J-larvae and that populations are univoltine (78–80, 84). Thus, model assumptions of a one-year life cycle in which the J-larva is the overwintering stage are likely valid for this region.

The model could potentially be improved by incorporating stage-specific temperature thresholds and a low temperature requirement for the completion of diapause development in the J-larval stage. Using different thresholds for immature stages (10°C for larvae and pupae, and 13.7°C for eggs) did not significantly affect prediction error, but the analysis was based on a limited observation dataset. Another area needing further study is whether termination or completion of diapause development in J-larvae requires a specific low temperature regime or other cue (110, 111). Recent work indicated that J-larvae may require at least two months at moderately low temperatures (*ca.* 12.8°C) to complete diapause development before pupation can begin (36, 64). However, future work should investigate whether J-larvae in southern populations have different thermal requirements to terminate diapause. A low temperature requirement for diapause completion was not added to the model in part because of DDRP’s single-year modeling structure, which prevents it from using climate data for the entire winter (e.g., November–February). The single-year modeling structure also prevents modeling a two-year life cycle for EAB.

In CONUS, trends towards earlier adult emergence over 20 recent years were predicted in some areas that experienced marked increases in average winter (December−February) temperatures (≥ 1.7°C) and spring (March−May) temperatures (0.5−3.4°C) since 1970, such as parts of New England and the South (112, 113). Similarly, trends in Europe occurred predominantly in areas (European Russia and Ukraine) that experienced rapid increases in winter and spring temperatures (0.07−0.08°C/year) between 1985 and 2020 (50). However, temporal trends in the DOY of first adult emergence were not significant for most areas of North America and Europe, which may be due to our use of a rather short (20 years) time-series dataset that likely exhibited serial correlation (i.e., the variable is correlated with itself over periods of time) (107). Alternatively, climate warming may not be impacting the timing of adult emergence as much as other phenological events. For example, a degree-day modeling study of EAB in Winnipeg, Canada concluded that climate change between 1970 and 2010 significantly impacted the date of peak activity but not the date of emergence in adults (73).

### 4.2 Climatic suitability model

The potential distribution of EAB based on 20-year climate averages overlapped with the ranges of all native *Fraxinus* species in North America and Europe, which is broadly consistent with previous climatic suitability models for EAB based on climate normals (41–47). Very high estimates of sensitivity of the climatic suitability model (99% of known presence records correctly modeled) suggest that it does not underpredict the potential distribution of EAB. However, we were unable to quantify model specificity (i.e., the proportion of known absence records correctly modeled) owing to a lack of data on absences of EAB in relation to climate. Thus, absence records are needed to assess whether the model overpredicts the potential distribution and to quantify overall model accuracy.

For certain years, cold stress excluded populations at the northern range of *Fraxinus* species in North America (i.e., green ash and black ash), which may be explained by severe cold events that can reduce overwintering survival rates in EAB (48, 54). While North America and Europe are experiencing warming winter temperatures and an overall decrease in the frequency and duration of extreme cold events (52, 106, 114), the frequency of high intensity cold events increased in some areas between 1950 and 2020 (115). Spatiotemporal variation in extreme cold events may explain why range-limiting cold stress for EAB between 2002 and 2021 in North America significantly decreased in some areas (e.g., in Quebec) but increased in others (e.g., in British Columbia). Thus, the impacts of climate change on EAB’s survival in the coldest parts of its distribution will likely vary across space and time.

Models based on 20-year climate averages indicated a minor role for heat stress in shaping EAB’s potential distribution in areas with native *Fraxinus* species. However, summer temperatures and the intensity, frequency, and duration of heat waves are increasing in western North America and are projected to worsen under enhanced global warming (49, 106, 116). Thus, the ranges of certain host species in southwestern North America such as *F. berlandieriana*, *F. greggii*, and *F. dipetala* could potentially become increasingly unsuitable under enhanced warming. Conversely, range-limiting heat stress for EAB did not significantly increase in the southeastern United States. Previous studies likely underestimated climatic suitability for EAB in this region because they used correlative algorithms and did not completely sample the species’ climatic distribution (42, 43).

Climatic suitability modeling in DDRP is based only on survival-limiting climate stresses, but the ability for a pest to complete its life cycle in a new climate also influences establishment risk [e.g.,(40)]. Insufficient degree-day accumulation for egg hatch to occur in parts of southern Canada and northern Europe during 2021, which had record-breaking summer temperatures, suggests that EAB is incapable of completing its life cycle in these areas. Similarly, models based on annual growing degree-days for Europe predicted that EAB could not complete development in many areas of Norway, Sweden, Finland, Ireland, and Great Britain (7, 46). Overlaying DDRP predictions of egg hatch produced for multiple years may provide greater insight into spatiotemporal variability in establishment risk based on life cycle completion. Future work could also investigate the relative roles of cold stress vs. incomplete life cycle progression in potentially excluding EAB from parts of southern Canada.

### 4.3 Potential sources of model error

Differences in monitoring methods used across studies likely explain at least some prediction error for adult phenological events. For example, some studies counted the number of exit holes to monitor adult emergence whereas others used trapping data. Adults are capable of immediate flight upon emergence (16); however, the efficiency in counting adults with traps and visual observation of adult exit holes may differ. While trap captures of EAB adults are affected by trap deployment location and many other factors such as beetle age and weather, visual observation of adult exit holes on infested ash trees may be less affected by those factors and thus may provide more accurate counts of emerging adults (J. Duan, unpublished data).

An additional explanation for prediction error for adult phenological events is our use of seven cohorts and a single set of cohort parameter values for all populations. These settings produced a distribution in adult emergence times that corresponded well with field data used for model calibration; however, distributions likely vary across the range of EAB. For example, decreasing the low bound for the timing of the completion of J-larvae (*xdist1*) may be appropriate for populations that have cohorts which require fewer degree days to complete J-larval development. The phenology model could be modified to accept a raster of values for cohort numbers and cohort parameter values; however, significantly more observations of pupal development and adult emergence of overwintered insects across latitudinal gradients are needed to estimate how J-larval development varies within populations across EAB’s range.

Differences between air temperatures and under-bark microclimates experienced by immature stages of EAB are a likely source of model error (32, 41, 117). Under-bark temperatures may be 1−7°C higher than ambient air temperatures during winter owing to the buffering capacity of snow and tree bark, potentially increasing rates of development and overwintering survival for EAB (117, 118). However, accounting for these temperature differences in our model is difficult because making assumptions of a constant level of thermal buffering are not valid owing to variability both within and between trees (117). For example, urban heating (45, 117) as well as variation in solar insolation within a forest patch (e.g., density of canopy cover) and within individual trees (e.g., the south side of a tree experiences warmer temperatures because it faces sunlight) can affect under-bark microclimates experienced by EAB (37, 66, 117, 118).

Our model assumes that tolerance to climate stress is constant across EAB’s range, but cold tolerance in EAB is a phenotypically plastic trait that varies both temporally and geographically (118–120). Mid-winter warm spells followed by an extreme cold event may have lethal effects on non-diapaused overwintering larvae owing to the breakdown of cryoprotectants during deacclimation, which decreases their cold tolerance (120). Populations in the northernmost (coldest) parts of EAB’s range may have higher survival rates during extreme cold events compared to southern populations because they acclimate to colder winter temperatures (119). A mechanistic model of overwintering mortality for EAB incorporated an equation to model the temperature dose-response relationship for overwintering J-larvae (48). However, the single-year modeling structure of DDRP would likely hinder using a similar method for the EAB model.

Biological factors not included in the model that may affect EAB’s development and survival include host tree health and nutritional quality of host tissues (66, 83, 121), presence of parasitoids and predators (122, 123), levels of host plant resistance (66, 124), and density of infestations (83). Phenology within a site may vary over time owing to host composition. Upon establishment, EAB kills the most susceptible ash species (green and black ash) first and then infests more resistant ash species (e.g., white ash, *Fraxinus americana* L.) and even white fringe trees (*Chionanthus virginicus* L.), which may result in delays in adult emergence in later years because of poorer host tree nutrition or other detrimental factors on larval development (125–127).

### 4.4 Applications for real-time decision support

The DDRP model for EAB has been operationalized for real-time decision support for CONUS at USPest.org (https://uspest.org/CAPS) and at the USA National Phenology Network (https://usanpn.org/data/forecasts/EAB). USPest.org provides all model outputs including phenological event maps for all life stages in raster (GeoTIFF) and summary map (PNG) formats. Conversely, the USA National Phenology Network presents predictions of first adult emergence and egg hatch as Pheno Forecasts (38) in summary map and interactive formats, and end users can sign up to receive e-mail notifications that provide advanced warnings (3, 2, and 1-week) of when these events will occur in their area. Interactive forecasts allow end users to zoom, pan, and interrogate maps (e.g., click on individual raster pixels in an area of interest). Pheno Forecasts for EAB show predictions only in climatically suitable areas and convert dates of phenological events to time relative to the map issue date (e.g., “This Week”, “Next Week”, etc.), which is a preferred format of many Pheno Forecast end users (38). Model outputs at both web sites are updated every three days. Climate datasets used for real-time modeling include PRISM daily data and daily-downscaled NMME (North American Multi-Model Ensemble) 7-month forecasts (128) at a spatial resolution of 4 km^2^ (55).

Real-time forecasts of adult emergence can support timely surveillance of adults, which are the most visible and therefore most easily detected life stage of the pest. For example, notifications that adults will emerge in three weeks can alert surveillance teams to finish installing detection devices such as sticky prism traps or funnel traps before adults begin flying in the canopy. Early detection of EAB is critical because infestations in ash trees are usually fatal, and failure to detect adults early may result in dispersal to new locations and increase the cost of ash treatments and removal (31, 129). Thus, early detection may help slow the spread of EAB in new regions such as the west coast of North America, where the pest was detected for the first time in 2022 in Forest Grove, Oregon (26). Forecasts of adult emergence may also improve the timing of systemic insecticide treatments and cover sprays that target adults feeding on host foliage (5, 130). For example, a manager can begin applying systemic insecticides when they know to expect adults in 2−3 weeks because females must feed on leaves for *ca.* 1−2 weeks before they begin laying eggs (5).

Real-time forecasts of egg hatch may help with the timing of insecticide applications that target EAB larvae, which are most effective when newly hatched larvae encounter the insecticide as they chew through the bark and into the cambial tissue (5, 130). Additionally, forecasts of adult oviposition and egg hatch may help biocontrol practitioners identify the critical periods for releases of biological control agents against EAB, which may increase the likelihood that agents establish and grow in the target area. Releases of the egg parasitoid *Oobius agrili* Zhang and Huang (Hymenoptera: Encyrtidae) must be timed during the EAB oviposition period because eggs are only suitable for parasitism up to the development of the neonate host larva (30, 131). Conversely, the larval parasitoids such as *Spathius agrili* Yang (Hymenoptera: Braconidae) and *S. galinae* Belokobylskij & Strazanac (Hymenoptera: Braconidae) must be released when 3^rd^ and 4^th^ instar larvae are present (131, 132).

While the DDRP model for EAB exhibited overall good performance, it should still be used conservatively for decision-making, particularly given that model validation analyses were based on a small observation dataset. As mentioned previously, surveillance and management activities could be implemented several days (e.g., a week) prior to the date of a predicted event. With additional observations of EAB, one could assess how to interpret forecasts in order to maximize the probability that adults are captured upon first emergence or that insecticides or biocontrol treatments are employed within the optimal window of time. To potentially avoid under-predicting the risk of establishment, the potential distribution could be defined as areas not under severe climate stress as opposed to defining it using both stress levels.

A lack of near real-time daily climate datasets for Canada and Europe would likely hinder using the model for real-time decision support in these regions despite a need for additional tools to manage EAB (12, 133). Continued spread of EAB in Canada is predicted to cause substantial economic impacts primarily owing to its destruction of urban forests (17). In Europe, the pest has spread to 16 regions of European Russia and to the east of Ukraine (19, 21, 28) and is expected to expand into neighboring countries within 5−20 years (27, 134). Declines in Europe’s most widespread ash species, European ash (*F. excelsior* L.), would have severe ecological and economic consequences because this species is a key component of many forests and has been widely planted in cities, parks and along roads as shade or ornamental trees (12, 21, 134, 135).

Future work on the EAB model could add capabilities that would allow it to predict EAB’s spread across the landscape and forecast the timing of its arrival (136, 137), which would provide a more comprehensive assessment of both where and when to expect this pest. For example, surveillance could be focused on areas that are climatically suitable for EAB and have a high likelihood of being colonized in the next five years. Within areas at highest risk of establishment, forecasts of adult emergence over subsequent years may help ensure that surveillance teams install detection devices on time.

## 5 Conclusions

We presented a spatialized model of phenology and climatic suitability for EAB for use in DDRP platform, which serves as an open-source decision support tool to help detect, monitor, and manage invasive threats. Real-time forecasts of adult emergence and egg hatch are particularly relevant for surveillance and for managing existing populations with pesticide treatments and parasitoid introductions. Model predictions based on historical climate data or future climate scenarios may provide insight into shifts in EAB’s phenology and potential distribution driven by climate change. Overall, the model exhibited strong performance, but additional monitoring data collected from across a latitudinal range are needed to further evaluate and potentially improve upon the model. Significant temporal trends towards earlier adult emergence, declines in range-limiting cold stress, and increases in range-limiting heat stress for certain regions suggest that climate change is influencing rates of development and survival in EAB in parts of its invaded range. Climate stresses were insufficient to exclude the pest from areas with native *Fraxinus* species in these continents; however, extreme weather events, climate warming, and an inability for EAB to complete its life cycle may reduce suitability for some areas.

## Data availability statement

Presence records used for climatic suitability modeling (https://doi.org/10.5281/zenodo.7493142) and the version of DDRP and species parameter file used for this study (https://doi.org/10.5281/zenodo.8018595) were archived in Zenodo. The most recent code for DDRP is available at GitHub (https://github.com/bbarker505/ddrp_v2.git).

## Author contributions

BB led the writing of the manuscript, compiled, and analyzed data, prepared figures and results, and helped secure funding. LC compiled and analyzed data, edited the manuscript, and helped secure funding. JD and TP collected data and edited the manuscript. All authors contributed to the article and approved the final submission.
