# Supplementary material for: An integrative phenology and climatic suitability model for emerald ash borer
Source: Front Insect Sci. 2023 Aug 29;3:1239173. doi: 10.3389/finsc.2023.1239173 (PMC10926479; doi:10.3389/finsc.2023.1239173)

***Supplementary Material***

**An integrative phenology and climatic suitability model for emerald ash borer**

**Brittany S. Barker^1,2*^, Leonard Coop^1,2^ , Jian J. Duan^3^, and Toby R. Petrice^4^**

^1^Oregon Integrated Pest Management Center, 4575 Research Way, Oregon State University, Corvallis, OR, United States

^2^Department of Horticulture, Oregon State University, 4017 Agriculture and Life Sciences Building, Oregon State University, Corvallis, OR, United States

^3^United States Department of Agriculture (USDA) Agricultural Research Service, Beneficial Insects Introduction Research Unit, Newark, DE, United States

^4^United States Department of Agriculture (USDA) Forest Service, Northern Research Station, Lansing, MI, United States

*** Correspondence:**Brittany Barker
brittany.barker@oregonstate.edu

# Supplementary Data 1

## Estimation of life stage duration parameters in the DDRP model for emerald ash borer

This section describes how we derived lower developmental thresholds (Tlow) and degree-day (DD) requirements for four life stages of emerald ash borer (EAB). When temperature-development data were available, we derived DD requirements by conducting a linear regression analysis on the data and adding a point to force the x-intercept to a common integer value in degrees Fahrenheit (54 °F, 12.2 °C). Other model parameter values remained constant in analyses that compared model predictions using different Tlow or DD requirement values (egg and larval stages). Daily estimates of minimum and maximum temperature (*T_min_* and *T_max_*, respectively) from the PRISM database at a spatial resolution of 4 km^2^ (<https://prism.oregonstate.edu>) (1) were used as climatic inputs.

### Overwintered J-larvae and pupae

We could not find any well-documented studies on the development of J-larvae (after a chilling period) and pupae as separate life stages. A study of male and female insects collected from Essex County, Ontario, reported an average Tlow of 11.75 °C for J-larvae collected in winter after a chilling period and an average Tlow of 14.15 °C for pupae (2). However, we could not assess potential data quality or re-analyze data from this study because it did not provide any information on sample sizes, rearing temperatures, or developmental rates at each temperature. A Tlow of 12.2 °C for both life stages is a compromise between the reported Tlow for each stage (i.e., 11.75 and 14.15 °C), and it also seems appropriate given that diapausing J-larvae require a period of ≥2 months at a temperature at *ca.* 12.8 C to resume development (3).

We estimated DD requirements for pupae as 135 DDs by converting days required for pupation in Tianjin, China (4) to DD units [20 days × (19 – 12.2 °C) = 136 DDs]. This value is similar to our re-calculation of DD requirements for pupae (142 DDs) based on data reported by Lyons and Jones (2). This involved calculating the number of days required for pupal development at 25 °C using Lyons and Jones’s (2) Tlow and DD requirements for pupae [15 DDs/(25 °C − 14.7 °C) = 11.1 days], and then re-calculating DD requirements using a Tlow of 12.2 °C [11.1 days × (25 – 12.2 °C) = 142 DDs].

### Adults

We conducted a linear regression analysis of data on the longevity of adult oviposition at four temperatures (5), which resulted in a Tlow of 16.7 °C (Supplementary Figure S1). This value is substantially higher than the Tlow of 11.85 °C for teneral adults (average for males and females) reported by Lyons and Jones (2). In the case of adults, which possibly have an “activity threshold” closer to 16.7 °C than to 12.2 °C, we should point out that mid-summers in temperate regions such as North America generally exceed both these values on a daily basis, so the Tlow for the adult stage should not be critical in determining model performance.

Adding a point to force the x-intercept to 12.2 °C resulted in a DD requirement of 289 DDs for 100% adult oviposition (adult emergence to oviposition termination). DD requirements for the adult stage (145 DDs) were estimated as 50% of oviposition (289 DDs × 0.5), or 145 DDs (Supplementary Figure S1). DD requirements for first oviposition (72 DDs) was estimated as 25% of oviposition (289 DDs × 0.25), which is similar to our re-calculation of DD requirements for the teneral adult period (55 DDs) reported by Lyons and Jones (2). This involved calculating the number of days required for development of teneral adults at 25 °C using Lyons and Jones’s (2) Tlow and DD requirements for this stage [179 DDs/(25 °C – 10.1 °C) = 4.3 days], and then re-calculating DD requirements using a Tlow of 12.2 °C [4.3 days × (25 – 12.2 °C) = 55 DDs].

### Egg and larval stages

We conducted a linear regression analysis of temperature-development data for eggs and larvae that were derived from insects in infested green ash (*Fraxinus pennsylvanica* Marshall) from Maryland, as presented in Tables 1 and 2 in Duan *et al.* (6). This analysis resulted in a Tlow of 13.8 °C for the egg stage (Supplementary Figure S2), which is consistent with the Tlow (13.9 °C) for this stage reported by Lyons and Jones (2). However, using a Tlow of 12.2 °C for the egg stage appears to be appropriate because the mean absolute error between predicted and observed dates of egg hatch (Supplementary Table S4) was only one day lower (12.7 days vs. 13.7 days) when using a model that applied a Tlow of 13.8 °C (stage duration = 159 DDCs) compared to the model that applied a Tlow of 12.2 °C (stage duration = 174 DDCs).

The development time for larvae (egg hatch to formation of J-larvae) was obtained by subtracting the egg development time [Table 1 in Duan *et al.* (6)] from the total development time for adult oviposition to mature J-larvae development [Table 2 in Duan *et al.* (6)]. In the linear regression analysis of these data, adding a point to force the x-intercept to 12.2 °C resulted in a DD requirement of 488 DDCs (Supplementary Figure S3). This value was increased because the model underpredicted dates of first J-larval development by an average of 47 days (range = 16−94 days, N = 6 observations; Supplementary Table S4). We increased the DD requirement in increments of 50 DDC until at least one prediction was nearly equal to the observed date, which reduced underprediction to an average of 10 days (range = 2−80 days). This resulted in a DD requirement of 700 DDCs.

# Supplementary Data 2

Previous phenology models for EAB have used a Tlow of 10 °C to predict adult activities. For example, Herms *et al.* (4) proposed that the emergence of EAB adults begins at 450−500 DDs based on a Tlow of 50 °F (i.e., 250−278 DDCs based on a Tlow of 10 °C) and starting date of January 1 (7). We modified the final DDRP model to test whether Herms *et al*.’s (7) parameters could produce more accurate predictions of first adult emergence by setting the Tlow of J-larvae and pupae to 10 °C and specifying a total of 250 DDC for these stages to complete development. Predictive accuracy was assessed using 30 observations of first adult emergence (Supplementary Table S4). According to this analysis, the final DDRP model produced a slightly lower mean absolute error than the model based on Herms *et al.*’s (4) parameters (7.5 days vs. 8.3 days). This finding suggests that a Tlow of 12.2 °C for larvae and pupae is appropriate.

# Supplementary Data 3

# Estimation of cohort parameters in the DDRP model for emerald ash borer

This section describes the field data and analyses that were conducted to help estimate DDRP model parameters that affect the timing of the completion of J-larvae across cohorts (hereafter, cohort parameters) in the DDRP model for EAB. These parameters include the shape of the distribution of completion times (*distro*_*shape*), and the mean (*distro_mean*), variance (*distro_var*), and the low bound and high bound (*xdist1* and *xdist2*, respectively) of completion times.

### Field data analysis

We used a monitoring dataset that characterized adult emergence patterns of EAB at six locations spanning a 500 km latitudinal gradient in the northcentral United States in 2011 and 2012 to calibrate cohort parameters. For each location and year, Duarte (8) collected data on 10, 25, 50, 75, 95, and 100% of the total emergence of EAB by counting the number of new exit holes weekly from first emergence until three consecutive weeks of no new emergence. DD accumulation from January 1 to the day of year for each emergence event was calculated using daily *T_min_* and *T_max_* data from nearby (<15 km) weather stations. DDs were computed using the modified sine wave method and a Tlow of 10 °C (50 °F).

We used a custom R script to re-calculate DD accumulation from Duarte’s (8) emergence data to account for the lower and upper developmental thresholds used in the DDRP model for EAB (12.2 °C and 36 °C, respectively). Following Duarte (8), we used a start date of January 1 and obtained daily *T_min_* and *T_max_* data from the National Climatic Data Center (NCDC) and Ohio Agricultural and Development Center weather system. Data for Delaware, OH were obtained from the Visual Crossing weather database because they were no longer available in the NCDC database. DDs were computed using the single triangle calculation method with upper threshold (9), which produces similar results as more complex sine-curve calculation methods (10). The results of this analysis are presented in Supplementary Table S1. The mean, minimum, and maximum values of DD accumulations at first, 50%, and 95% adult emergence were summarized across all locations and years (Supplementary Table S2) to help calibrate cohort parameters as described below. Additionally, line plots were created to visualize the shapes and distributions of adult emergence times (Supplementary Figure S4).

### Cohort parameter calibration

We tested among multiple combinations of five cohort parameter values (Supplementary Table S3) after identifying appropriate ranges of values for each parameter as follows. Reasonable DD values in Celsius units (DDC, Tlow = 12.2 °C) for *xdist1*, *distro_mean,* and *xdist2* were identified by subtracting the duration of the pupal stage (135 DDC) from summaries of DDCs at first, 50%, and 95% adult emergence estimated from Duarte’s (8) data (Supplementary Table S2). Based on these results, we tested values from 30 to 90 DDCs in increments of 5 DDC for *xdist1,* from 120 to 220 DDCs in increments of 5 DDC for *distro_mean*, and from 300 to 1000 DDCs in increments of 50 DDC for *xdist2*. We tested values from 15000 to 50000 for the *distro_var* parameter in increments of 5000.

A matrix of all possible combinations of parameter values was generated and then filtered to remove combinations that poorly matched summarized field data (Supplementary Table S3). First, we retained only combinations in which 45–60% of the population had completed development prior to the *distro_mean* value so that the model-predicted timing of 50% adult emergence aligned well with field measurements of 50% of total adult emergence. Second, we retained combinations in which the first cohort represented < 20% of the total population because initial emergence of EAB in the field consists of only a few individuals out of the total population (i.e., a low proportion of the total population). Models with nearly identical parameter combinations were removed by rounding DDC values to the nearest 5 and retaining the model in which the percentage of the population that had completed development prior to the *distro_mean* was closest to 50%.

The parameter combination that resulted in the lowest mean absolute error between the predicted and observed days of the year (DOYs) of first and 50% adult emergence was used in the final DDRP model (parameter combination 15, Supplementary Table S3). First and 50% adult emergence were estimated as the earliest DOY and weighted mean of DOYs for adult emergence across cohorts, respectively.

All models applied the “lognormal” option for the *distro_shape* parameter to account for a positive skew in adult emergence times across most locations and years (Supplementary Figure S4). The application of seven cohorts combined with the final cohort parameter values resulted in distribution of adult emergence times that corresponded well with field data (Supplementary Figure S4). Preliminary analyses indicated that increasing the number of cohorts (nine and 11 were tested) produced similar distributions, which resulted in very similar predictions as the seven-cohort model. Thus, we chose to apply seven cohorts to keep model run times reasonable (computational load increases with an increasing number of cohorts).

**REFERENCES**

1. Daly C, Halbleib H, Smith J, Gibson W, Doggett M, Taylor G, Curtis J, Pasteris P. Physiographically sensitive mapping of climatological temperature and precipitation across the conterminous United States. *Int J Climatol* (2008) 28:2031–64. doi: 10.1002/joc.1688

2. Lyons DB, Jones GC. The biology and phenology of emerald ash borer. In: Gottschalk KW, editor. *Proceedings, 16th U.S. Department of Agriculture interagency research forum on gypsy moth and other invasive species; 2005 Jan 18‒21; Annapolis, MD; GTR- NE-337*. Newtown Square, PA: U.S. Department of Agriculture, Forest Service, Northeastern Research Station (2005). p. 62–63.

3. Duan JJ, Schmude JM, Larson KM. Effects of low temperature exposure on diapause, development, and reproductive fitness of the emerald ash borer (Coleoptera: Buprestidae): implications for voltinism and laboratory rearing. *J Econ Entomol* (2021) 114:201–8. doi: 10.1093/jee/toaa252

4. Wang XY, Cao LM, Yang ZQ, Duan JJ, Gould JR, Bauer LS. Natural enemies of emerald ash borer (Coleoptera: Buprestidae) in northeast China, with notes on two species of parasitic Coleoptera. *Can Entomol* (2016) 148:329–42. doi: 10.4039/tce.2015.57

5. Petrice TR, Bauer LS, Miller DL, Poland TM, Ravlin FW. A phenology model for simulating *Oobius agrili* (Hymenoptera: Encyrtidae) seasonal voltinism and synchrony with emerald ash borer oviposition. *Environ Entomol* (2020) 50:280–92. doi: 10.1093/ee/nvaa169

6. Duan JJ, Watt TIM, Taylor P, Larson K. Effects of ambient temperature on egg and larval development of the invasive emerald ash borer (Coleoptera: Buprestidae): implications for laboratory rearing. *For Entomol* (2013) 106:2101–8. doi: 10.1603/ec13131

7. Herms DA, McCullough DG, Clifford CS, Smitley DR, Miller FD, Cranshaw W. *Insecticide options for protecting ash trees from emerald ash borer, North Central IPM Center Bulletin*, 3rd Edition. Urbana, IL: National IPM Center (2019). Available at: https://extension.entm.purdue.edu/EAB/PDF/NC-IPM.pdf

8. Duarte S. *Characterizing prepupal diapause and adult emergence phenology of emerald ash borer.* Columbus, OH: Ohio State University (2013). Master’s thesis.

9. Sevacherian V, Stern VM, Mueller AJ. Heat accumulation for timing *Lygus* control measures in a safflower-cotton complex. *J Econ Entomol* (1977) 70:399–402. doi: 10.1093/jee/70.4.399

10. Roltsch WJ, Zalom A, Strand JF, Pitcairn MJ, Frank JR, Ann GZ. Evaluation of several degree-day estimation methods in California climates. *Int J Biometeorol* (1999) 42:169–76. doi: 10.1007/s004840050101

# Supplementary Figures and Tables

## Supplementary Tables

**Table S1.** Degree-day accumulations for adult emergence events estimated using a lower developmental threshold (Tlow) of 10 °C compared to a Tlow of 12.2 °C.

|  |  | 2011 | | | 2012 | |  |
| --- | --- | --- | --- | --- | --- | --- | --- |
| Location | Event | Tlow=10°C | Tlow=2.2°C | Tlow=10°C | | Tlow=12.2°C | |
| Cincinnati OH | first | 334 | 217 | 265 | | 162 | |
| Cincinnati OH | 10% | 370 | 246 | 385 | | 255 | |
| Cincinnati OH | 25% | 463 | 321 | 435 | | 283 | |
| Cincinnati OH | 50% | 541 | 386 | 503 | | 342 | |
| Cincinnati OH | 75% | 641 | 470 | 650 | | 460 | |
| Cincinnati OH | 95% | 871 | 602 | 1047 | | 796 | |
| Cincinnati OH | 100% | 1508 | 1214 | 1586 | | 1262 | |
| Columbus OH | first | - | - | - | | - | |
| Columbus OH | 10% | - | - | - | | - | |
| Columbus OH | 25% | - | - | 411 | | 281 | |
| Columbus OH | 50% | - | - | 415 | | 284 | |
| Columbus OH | 75% | - | - | 479 | | 334 | |
| Columbus OH | 95% | - | - | 804 | | 603 | |
| Columbus OH | 100% | - | - | 1253 | | 991 | |
| Delaware OH | first | 350 | 228 | 182 | | 102 | |
| Delaware OH | 10% | 358 | 240 | 387 | | 253 | |
| Delaware OH | 25% | 433 | 318 | 512 | | 355 | |
| Delaware OH | 50% | 461 | 318 | 562 | | 394 | |
| Delaware OH | 75% | 489 | 364 | 692 | | 501 | |
| Delaware OH | 95% | 828 | 640 | 1338 | | 961 | |
| Delaware OH | 100% | 1263 | 1011 | 1852 | | 1418 | |
| Midland MI | first | - | - | 367 | | 235 | |
| Midland MI | 10% | - | - | 401 | | 255 | |
| Midland MI | 25% | - | - | 410 | | 262 | |
| Midland MI | 50% | 373 | 254 | 496 | | 328 | |
| Midland MI | 75% | 450 | 312 | 597 | | 413 | |
| Midland MI | 95% | 519 | 365 | 946 | | 711 | |
| Midland MI | 100% | 924 | 713 | 1270 | | 965 | |
| Toledo OH | first | - | - | 287 | | 176 | |
| Toledo OH | 10% | 292 | 168 | 322 | | 201 | |
| Toledo OH | 25% | 392 | 242 | 404 | | 262 | |
| Toledo OH | 50% | 493 | 313 | 482 | | 320 | |
| Toledo OH | 75% | 578 | 366 | 544 | | 364 | |
| Toledo OH | 95% | 911 | 605 | 738 | | 517 | |
| Toledo OH | 100% | 1510 | 1058 | 1496 | | 1130 | |
| Wooster OH | first | 341 | 237 | - | | - | |
| Wooster OH | 10% | 402 | 286 | 352 | | 233 | |
| Wooster OH | 25% | 418 | 299 | 376 | | 250 | |
| Wooster OH | 50% | 458 | 332 | 420 | | 287 | |
| Wooster OH | 75% | 475 | 343 | 500 | | 347 | |
| Wooster OH | 95% | 603 | 446 | 594 | | 505 | |
| Wooster OH | 100% | 1198 | 948 | 1529 | | 1200 | |

**Table S2.** Summary statistics (mean, minimum, and maximum) for adult emergence events in emerald ash borer and corresponding estimates of the end of J-larval development in degree-day units (DDC) based a lower development threshold of 12.2 °C. Statistics for the end of J-larval development were calculated by subtracting degree-day requirements for the pupal stage (135 DDC) from corresponding statistics for first, 50%, and 95% of total adult emergence. This information was used to identify appropriate ranges of values for five cohort parameters.

|  | Adult emergence (DDC) | | |  | End of J-larval development (Adult emergence DDC – 135 DDC) | | | | |
| --- | --- | --- | --- | --- | --- | --- | --- | --- | --- |
| **Event** | **Mean** | **Min** | **Max** | | |  | **Mean** | **Min** | **Max** |
| First emergence | 194 | 102 | 237 | | |  | 59 | − | 102 |
| 50% of total emergence | 323 | 254 | 394 | | |  | 188 | 119 | 259 |
| 95% of total emergence | 614 | 365 | 961 | | |  | 479 | 230 | 826 |

**Table S3.** Cohort parameter values in degree-day (DD) units (lower developmental threshold = 12.2 °C) that were tested to identify the DDRP model that minimized prediction error for first and peak adult emergence in emerald ash borer. Parameters include the low bound, mean, high bound, and variance of development completion times for J-larvae (*xdist1*, *distro_mean*, *xdist2*, and *distro_var*, respectively). The end of J-larval development in DD units is shown for the first cohort, weighted mean across cohorts, and last cohort for each parameter combination (rounded to the nearest 5). Bold font indicates the combination used in the final DDRP model.

|  | Parameters | | | |  | End of J-larval development | | |  | Mean absolute error | | |
| --- | --- | --- | --- | --- | --- | --- | --- | --- | --- | --- | --- | --- |
| combo | *xdist1* | *distro_mean* | *xdist2* | *distro_var* |  | first cohort | weighted mean | last cohort |  | first adult emergence | peak adult emergence | sum |
| 1 | 30 | 190 | 350 | 15000 |  | 60 | 160 | 325 |  | 5 | 4.9 | 9.9 |
| 2 | 30 | 210 | 400 | 15000 |  | 70 | 185 | 370 |  | 4.4 | 4.7 | 9.1 |
| 3 | 30 | 215 | 400 | 20000 |  | 65 | 180 | 370 |  | 4.2 | 4.5 | 8.7 |
| 4 | 30 | 215 | 400 | 15000 |  | 70 | 190 | 370 |  | 4.2 | 4.7 | 8.9 |
| 5 | 30 | 215 | 550 | 15000 |  | 80 | 205 | 490 |  | 3.7 | 5.1 | 8.8 |
| 6 | 35 | 190 | 350 | 15000 |  | 60 | 160 | 325 |  | 4.5 | 4.9 | 9.4 |
| 7 | 35 | 210 | 400 | 15000 |  | 70 | 185 | 370 |  | 4.3 | 4.7 | 9 |
| 8 | 35 | 220 | 550 | 15000 |  | 85 | 210 | 490 |  | 3.9 | 5.3 | 9.2 |
| 9 | 40 | 220 | 400 | 15000 |  | 75 | 195 | 370 |  | 3.8 | 4.9 | 8.7 |
| 10 | 40 | 220 | 550 | 15000 |  | 85 | 210 | 490 |  | 3.9 | 5.2 | 9.1 |
| 11 | 45 | 195 | 350 | 15000 |  | 70 | 165 | 325 |  | 4.4 | 4.8 | 9.2 |
| 12 | 50 | 215 | 400 | 15000 |  | 80 | 190 | 370 |  | 3.8 | 4.7 | 8.5 |
| 13 | 50 | 220 | 400 | 15000 |  | 80 | 195 | 370 |  | 3.8 | 4.8 | 8.6 |
| 14 | 55 | 200 | 350 | 15000 |  | 80 | 170 | 325 |  | 4 | 4.7 | 8.7 |
| **15** | **60** | **200** | **350** | **15000** |  | **80** | **175** | **325** |  | **3.7** | **4.6** | **8.3** |
| 16 | 60 | 220 | 400 | 15000 |  | 85 | 195 | 370 |  | 3.8 | 4.9 | 8.7 |
| 17 | 65 | 205 | 350 | 15000 |  | 85 | 180 | 325 |  | 4 | 4.6 | 8.6 |
| 18 | 65 | 220 | 400 | 15000 |  | 90 | 195 | 370 |  | 4.1 | 5.1 | 9.2 |
| 19 | 75 | 210 | 350 | 15000 |  | 95 | 185 | 325 |  | 4.3 | 4.5 | 8.8 |
| 20 | 80 | 210 | 350 | 15000 |  | 100 | 185 | 325 |  | 4.5 | 4.8 | 9.3 |
| 21 | 85 | 215 | 350 | 15000 |  | 105 | 190 | 330 |  | 5.3 | 4.8 | 10.1 |
| 22 | 90 | 215 | 350 | 15000 |  | 110 | 195 | 330 |  | 5.6 | 4.7 | 10.3 |

**Table S4.** Field observations of phenology used to calibrate and validate (Set = cal and val, respectively) the DDRP model for emerald ash borer. The geographic origin, event type, date, day of year (DOY_obs_), and source (Ref) of each observation is provided. Differences in days between model-predicted DOY (DOY_mod_) and DOY_obs_ (Diff = predicted DOY– observed DOY) were calculated for pupation of overwintered larvae, emergence of overwintered adults, egg-laying, and egg hatch in the model (coded in the model as l0, p0, a0, and e1, respectively). Observations of first J-larval development (coded in the model as l1) were used to calibrate the degree-day requirements for the larval stage (instar 1 to J-larval development). Predictions for locations that lacked precise coordinate data were obtained by averaging predictions in polygons of the city. A polygon for Detroit, MI was used as a substitute for a non-georeferenced location in the Windsor airport area of Essex County, Ontario in Canada (Ref = 14) owing to a lack of PRISM climate data for that area.

| **Set** | **Country** | **State** | **City** | **Lat** | **Lon** | **Stage** | **Event** | **PEM** | **Year** | **Date** | **DOYobs** | **DOYpred** | **Diff** | **Ref** |
| --- | --- | --- | --- | --- | --- | --- | --- | --- | --- | --- | --- | --- | --- | --- |
| cal | United States | MI | Midland | 43.624 | –84.247 | Adult | first adult emerg. | p0 | 2011 | 6/17/2011 | 168 | 165 | –3 | 1 |
| cal | United States | MI | Midland | 43.624 | –84.247 | Adult | first adult emerg. | p0 | 2012 | 5/31/2012 | 152 | 149 | –3 | 1 |
| cal | United States | OH | Cincinnati | 39.133 | –84.358 | Adult | first adult emerg. | p0 | 2011 | 5/20/2011 | 140 | 143 | 3 | 1 |
| cal | United States | OH | Cincinnati | 39.133 | –84.358 | Adult | first adult emerg. | p0 | 2012 | 4/25/2012 | 116 | 125 | 9 | 1 |
| cal | United States | OH | Columbus | 40.011 | –83.036 | Adult | first adult emerg. | p0 | 2012 | 5/10/2012 | 131 | 130 | –1 | 1 |
| cal | United States | OH | Delaware | 40.354 | –83.064 | Adult | first adult emerg. | p0 | 2011 | 6/2/2011 | 153 | 152 | –1 | 1 |
| cal | United States | OH | Delaware | 40.354 | –83.064 | Adult | first adult emerg. | p0 | 2012 | 5/25/2012 | 146 | 140 | –6 | 1 |
| cal | United States | OH | Toledo | 41.657 | –83.552 | Adult | first adult emerg. | p0 | 2011 | 5/30/2011 | 150 | 154 | 4 | 1 |
| cal | United States | OH | Toledo | 41.657 | –83.552 | Adult | first adult emerg. | p0 | 2012 | 5/12/2012 | 133 | 142 | 9 | 1 |
| cal | United States | OH | Wooster | 40.779 | –81.937 | Adult | first adult emerg. | p0 | 2011 | 6/1/2011 | 152 | 152 | 0 | 1 |
| cal | United States | OH | Wooster | 40.779 | –81.937 | Adult | first adult emerg. | p0 | 2012 | 5/18/2012 | 139 | 141 | 2 | 1 |
| cal | United States | MI | Midland | 43.624 | –84.247 | Adult | peak adult emerg. | p0 | 2011 | 6/18/2011 | 168 | 177 | 9 | 1 |
| cal | United States | MI | Midland | 43.624 | –84.247 | Adult | peak adult emerg. | p0 | 2012 | 6/16/2012 | 167 | 163 | –4 | 1 |
| cal | United States | OH | Cincinnati | 39.133 | –84.358 | Adult | peak adult emerg. | p0 | 2011 | 6/7/2011 | 157 | 152 | –5 | 1 |
| cal | United States | OH | Cincinnati | 39.133 | –84.358 | Adult | peak adult emerg. | p0 | 2012 | 5/24/2012 | 144 | 137 | –7 | 1 |
| cal | United States | OH | Columbus | 40.011 | –83.036 | Adult | peak adult emerg. | p0 | 2012 | 5/18/2012 | 138 | 144 | 6 | 1 |
| cal | United States | OH | Delaware | 40.354 | –83.064 | Adult | peak adult emerg. | p0 | 2011 | 6/9/2011 | 159 | 161 | 2 | 1 |
| cal | United States | OH | Delaware | 40.354 | –83.064 | Adult | peak adult emerg. | p0 | 2012 | 6/11/2012 | 162 | 150 | –12 | 1 |
| cal | United States | OH | Toledo | 41.657 | –83.552 | Adult | peak adult emerg. | p0 | 2011 | 6/14/2011 | 164 | 162 | –2 | 1 |
| cal | United States | OH | Toledo | 41.657 | –83.552 | Adult | peak adult emerg. | p0 | 2012 | 6/2/2012 | 153 | 153 | 0 | 1 |
| cal | United States | OH | Wooster | 40.779 | –81.937 | Adult | peak adult emerg. | p0 | 2011 | 6/12/2011 | 162 | 162 | 0 | 1 |
| cal | United States | OH | Wooster | 40.779 | –81.937 | Adult | peak adult emerg. | p0 | 2012 | 5/28/2012 | 148 | 152 | 4 | 1 |
| cal | United States | NC | Garner | 35.644 | –78.58 | Larva | first J–larval devel. | l1 | 2019 | 8/23/2019 | 235 | 200 | –35 | 2 |
| cal | United States | NC | Garner | 35.644 | –78.58 | Larva | first J–larval devel. | l1 | 2020 | 10/13/2020 | 287 | 207 | –80 | 2 |
| cal | United States | NC | Garner | 35.644 | –78.58 | Larva | first J–larval devel. | l1 | 2021 | 8/2/2021 | 214 | 209 | –5 | 2 |
| cal | United States | TN | Oak Ridge | 35.932 | –84.31 | Larva | first J–larval devel. | l1 | 2017 | 7/28/2017 | 209 | 207 | –2 | 3 |
| cal | United States | TN | Powell | 36.058 | –84.056 | Larva | first J–larval devel. | l1 | 2016 | 9/20/2016 | 264 | 212 | –52 | 3 |
| cal | United States | TN | Powell | 36.058 | –84.056 | Larva | first J–larval devel. | l1 | 2017 | 8/22/2016 | 235 | 214 | –21 | 3 |
| val | United States | NC | Garner | 35.644 | –78.58 | Pupa | first pupation | l0 | 2020 | 3/3/2020 | 63 | 68 | 5 | 2 |
| val | United States | NC | Garner | 35.644 | –78.58 | Pupa | first pupation | l0 | 2021 | 4/2/2021 | 92 | 87 | –5 | 2 |
| val | United States | NC | Granville | 36.153 | –78.768 | Pupa | first pupation | l0 | 2017 | 2/28/2017 | 59 | 85 | 26 | 4 |
| val | Russia | MOS | Moscow | 55.983 | 37.167 | Adult | first adult emerg. | p0 | 2013 | 6/8/2013 | 159 | 163 | 4 | 5 |
| val | Russia | MOS | Moscow | 55.983 | 37.167 | Adult | first adult emerg. | p0 | 2014 | 6/2/2014 | 153 | 158 | 5 | 5 |
| val | United States | DE | Newark | 39.668 | –75.742 | Adult | first adult emerg. | p0 | 2020 | 5/25/2020 | 162 | 156 | –6 | 6 |
| val | United States | GA | Suwanee | 34.027 | –84.049 | Adult | first adult emerg. | p0 | 2022 | 4/17/2022 | 107 | 115 | 8 | 7 |
| val | United States | LA | Andreas | 32.663 | −93.369 | Adult | first adult emerg. | p0 | 2016 | 4/4/2016 | 95 | 91 | –4 | 8 |
| val | United States | MI | Ann Arbor |  |  | Adult | first adult emerg. | p0 | 2003 | 6/9/2003 | 160 | 169 | 9 | 9 |
| val | United States | MI | Ashley | 43.234 | –84.448 | Adult | first adult emerg. | p0 | 2019 | 6/23/2019 | 174 | 174 | 0 | 10 |
| val | United States | MI | Eastport | 45.114 | –85.332 | Adult | first adult emerg. | p0 | 2016 | 6/23/2016 | 175 | 175 | 0 | 10 |
| val | United States | MI | Ithaca | 43.234 | –84.448 | Adult | first adult emerg. | p0 | 2016 | 6/8/2016 | 160 | 162 | 2 | 10 |
| val | United States | MI | Ithaca | 43.234 | –84.448 | Adult | first adult emerg. | p0 | 2017 | 6/8/2017 | 159 | 162 | 3 | 10 |
| val | United States | MI | Ithaca | 43.234 | –84.448 | Adult | first adult emerg. | p0 | 2018 | 6/5/2018 | 156 | 156 | 0 | 11 |
| val | United States | MI | Lansing | 42.697 | –84.375 | Adult | first adult emerg. | p0 | 2016 | 6/20/2016 | 172 | 162 | –10 | 10 |
| val | United States | MI | Lansing | 42.694 | –84.382 | Adult | first adult emerg. | p0 | 2016 | 6/20/2016 | 172 | 162 | –10 | 10 |
| val | United States | MI | Lansing | 42.697 | –84.375 | Adult | first adult emerg. | p0 | 2017 | 6/15/2017 | 166 | 161 | –5 | 10 |
| val | United States | MI | Lansing | 42.694 | –84.382 | Adult | first adult emerg. | p0 | 2017 | 6/2/2017 | 153 | 161 | 8 | 10 |
| val | United States | MI | Lansing | 42.697 | –84.375 | Adult | first adult emerg. | p0 | 2018 | 6/21/2018 | 172 | 153 | –19 | 10 |
| val | United States | MI | Lansing | 42.694 | –84.382 | Adult | first adult emerg. | p0 | 2018 | 6/14/2018 | 165 | 153 | –12 | 10 |
| val | United States | MI | Lansing | 42.697 | –84.375 | Adult | first adult emerg. | p0 | 2019 | 7/2/2019 | 183 | 171 | –12 | 11 |
| val | United States | MI | Lansing | 42.694 | –84.382 | Adult | first adult emerg. | p0 | 2019 | 6/25/2019 | 176 | 171 | –5 | 11 |
| val | United States | MI | Novi |  |  | Adult | first adult emerg. | p0 | 2004 | 5/15/2004 | 136 | 159 | 23 | 9 |
| val | United States | MI | Troy |  |  | Adult | first adult emerg. | p0 | 2004 | 5/28/2004 | 149 | 157 | 8 | 9 |
| val | United States | MI | Troy | 42.603 | –83.251 | Adult | first adult emerg. | p0 | 2020 | 5/29/2020 | 150 | 161 | 11 | 7 |
| val | United States | MI | Troy | 42.603 | –83.251 | Adult | first adult emerg. | p0 | 2021 | 5/18/2021 | 138 | 156 | 18 | 7 |
| val | United States | MI | Troy | 42.603 | –83.251 | Adult | first adult emerg. | p0 | 2022 | 5/31/2022 | 151 | 157 | 6 | 7 |
| val | United States | NC | Garner | 35.644 | –78.58 | Adult | first adult emerg. | p0 | 2021 | 4/15/2021 | 105 | 118 | 13 | 2 |
| val | United States | NY | Randolph | 42.162 | –78.975 | Adult | first adult emerg. | p0 | 2010 | 6/10/2010 | 161 | 157 | –4 | 12 |
| val | United States | NY | Rockland | 41.98 | –74.706 | Adult | first adult emerg. | p0 | 2021 | 6/28/2021 | 179 | 183 | 4 | 7 |
| val | United States | NY | Syracuse | 43.065 | –76.044 | Adult | first adult emerg. | p0 | 2016 | 6/14/2016 | 159 | 162 | 3 | 13 |
| val | United States | NY | Syracuse | 43.065 | –76.044 | Adult | first adult emerg. | p0 | 2017 | 6/14/2017 | 159 | 164 | 5 | 13 |
| val | United States | WI | Milwaukee | 42.986 | –88.184 | Adult | first adult emerg. | p0 | 2019 | 6/21/2019 | 172 | 177 | 5 | 7 |
| val | United States | DE | Newark | 39.668 | –75.742 | Adult | peak adult emerg. | p0 | 2020 | 6/4/2020 | 171 | 165 | –6 | 6 |
| val | United States | LA | Andreas | 32.663 | −93.369 | Adult | peak adult emerg. | p0 | 2016 | 4/29/2016 | 120 | 106 | –14 | 8 |
| val | United States | MI | Ann Arbor |  |  | Adult | peak adult emerg. | p0 | 2003 | 6/16/2003 | 167 | 180 | 13 | 9 |
| val | United States | MI | Detroit |  |  | Adult | peak adult emerg. | p0 | 2003 | 6/25/2003 | 176 | 179 | 3 | 14 |
| val | United States | MI | Detroit |  |  | Adult | peak adult emerg. | p0 | 2004 | 6/11/2004 | 163 | 166 | 3 | 14 |
| val | United States | MI | Novi |  |  | Adult | peak adult emerg. | p0 | 2004 | 6/5/2004 | 157 | 171 | 14 | 9 |
| val | United States | MI | Troy |  |  | Adult | peak adult emerg. | p0 | 2004 | 6/26/2004 | 178 | 168 | –10 | 9 |
| val | United States | MI | Whitmore | 42.381 | –83.763 | Adult | peak adult emerg. | p0 | 2005 | 6/24/2005 | 175 | 171 | –4 | 15 |
| val | United States | DC | DC | 38.906 | –76.973 | Adult | peak adult activity | a0 | 2017 | 6/9/2017 | 160 | 148 | –12 | 16 |
| val | United States | MI | Eastport | 45.114 | –85.332 | Adult | peak adult activity | a0 | 2016 | 7/7/2016 | 189 | 196 | 7 | 10 |
| val | United States | MI | Ithaca | 43.234 | –84.448 | Adult | peak adult activity | a0 | 2016 | 7/5/2016 | 187 | 182 | –5 | 10 |
| val | United States | MI | Ithaca | 43.234 | –84.448 | Adult | peak adult activity | a0 | 2017 | 6/15/2017 | 166 | 180 | 14 | 10 |
| val | United States | MI | Ithaca | 43.234 | –84.448 | Adult | peak adult activity | a0 | 2018 | 6/21/2018 | 172 | 176 | 4 | 10 |
| val | United States | MI | Ithaca | 43.234 | –84.448 | Adult | peak adult activity | a0 | 2019 | 7/16/2019 | 197 | 189 | –8 | 11 |
| val | United States | MI | Lansing | 42.808 | –84.359 | Adult | peak adult activity | a0 | 2006 | 7/10/2006 | 191 | 181 | –10 | 17 |
| val | United States | MI | Lansing | 42.808 | –84.359 | Adult | peak adult activity | a0 | 2007 | 6/15/2007 | 166 | 173 | 7 | 17 |
| val | United States | MI | Lansing | 42.697 | –84.375 | Adult | peak adult activity | a0 | 2016 | 7/4/2016 | 186 | 180 | –6 | 10 |
| val | United States | MI | Lansing | 42.694 | –84.382 | Adult | peak adult activity | a0 | 2016 | 6/20/2016 | 172 | 180 | 8 | 10 |
| val | United States | MI | Lansing | 42.697 | –84.375 | Adult | peak adult activity | a0 | 2017 | 7/5/2017 | 186 | 177 | –9 | 10 |
| val | United States | MI | Lansing | 42.694 | –84.382 | Adult | peak adult activity | a0 | 2017 | 6/22/2017 | 173 | 177 | 4 | 10 |
| val | United States | MI | Lansing | 42.694 | –84.382 | Adult | peak adult activity | a0 | 2018 | 7/5/2018 | 186 | 174 | –12 | 10 |
| val | United States | MI | Lansing | 42.697 | –84.375 | Adult | peak adult activity | a0 | 2018 | 6/21/2018 | 172 | 174 | 2 | 10 |
| val | United States | MI | Lansing | 42.694 | –84.382 | Adult | peak adult activity | a0 | 2019 | 7/9/2019 | 190 | 187 | –3 | 11 |
| val | United States | MI | Lansing | 42.697 | –84.375 | Adult | peak adult activity | a0 | 2019 | 7/2/2019 | 183 | 187 | 4 | 11 |
| val | United States | TN | Oak Ridge | 35.932 | –84.31 | Larva | first egg hatch | e1 | 2017 | 6/22/2017 | 173 | 150 | –23 | 3 |
| val | United States | TN | Powell | 36.058 | –84.056 | Larva | first egg hatch | e1 | 2016 | 6/1/2016 | 153 | 162 | 9 | 3 |
| val | United States | TN | Powell | 36.058 | –84.056 | Larva | first egg hatch | e1 | 2017 | 6/13/2017 | 164 | 155 | –9 | 3 |

**References**

1. Duarte S. *Characterizing prepupal diapause and adult emergence phenology of emerald ash borer.* Columbus, OH: Ohio State University (2013). Master’s thesis.
2. Bohannon GR, Johnson CL, Jetton RM, Oten KLF. Behavioral ecology phenology and voltinism of emerald ash borer (Coleoptera: Buprestidae) in central North Carolina. *Environ Entomol* (2022) 51:1077–85. doi: 10.1093/ee/nvac088
3. Palmer JF. *Biological control of emerald ash borer in the southern U.S.: seasonality, phenological synchrony, and implications for management*. Knoxville, TN: University of Tennessee (2018). Master’s thesis.
4. Nalepa CA, Oten KLF, Bertone MA. Overwintering developmental stages of emerald ash borer in North Carolina. *Florida Entomol* (2021) 104:213–17. doi: 10.1653/024.104.0310
5. Orlova-Bienkowskaja MJ, Bieńkowski AO. The life cycle of the emerald ash borer *Agrilus planipennis* in European Russia and comparisons with its life cycles in Asia and North America. *Agric For Entomol* (2016) 18:182–88. doi: 10.1111/afe.12140
6. Duan JJ. Data collected for this study.
7. Natures Notebook Database. 2022. Data download using the *rnpn* R package v. 1.25 [Accessed Nov 10, 2022].
8. Kaur B. *Ecology of emerald ash borer (Agrilus planipennis: Buprestidae) in Louisiana*. Baton Rouge, LA: Louisiana State University (2018). Master’s thesis.
9. Brown-Rytlewski DE, Wilson MA. Tracking the emergence of emerald ash borer adults. In: Mastro V, Reardon R, editors. *Emerald Ash Borer Research and Technology Development Meeting; 2005 Oct 5‒6; Romulus, MI; FHTET-2004-15*. U.S. Department of Agriculture, Forest Service, Morgantown, WV (2005). p. 13‒14.
10. Petrice TR. Data collected for this study.
11. Petrice TR, Bauer LS, Miller DL, Poland TM, Ravlin FW. A phenology model for simulating *Oobius agrili* (Hymenoptera: Encyrtidae) seasonal voltinism and synchrony with emerald ash borer oviposition. *Environ Entomol* (2020) 50:1–13. doi: 10.1093/ee/nvaa169
12. Fierke MK, Whitmore MC, Foelker C, Vandenberg JD, Carlson J. Delimitation and management of emerald ash borer (Coleoptera: Buprestidae): Case study at an outlier infestation in southwestern New York State, United States of America. *Can Entomol* (2013) 145:577–87. doi: 10.4039/tce.2013.39
13. Jones MI, Gould JR, Mahon HJ, Fierke MK, Sullivan B. Phenology of emerald ash borer (Coleoptera: Buprestidae) and its introduced larval parasitoids in the northeastern United States. *J Econ Entomol* (2020) 113:622–32. doi: 10.1093/jee/toz304
14. Lyons DB, Jones GC. The biology and phenology of emerald ash borer. In: Gottschalk KW, editor. *Proceedings, 16th U.S. Department of Agriculture interagency research forum on gypsy moth and other invasive species; 2005 Jan 18‒21; Annapolis, MD; GTR- NE-337*. Newtown Square, PA: U.S. Department of Agriculture, Forest Service, Northeastern Research Station (2005). p. 62–63.
15. Rodriguez-Saona CR, Miller JR, Poland TM, Kuhn TM, Otis GW, Turk T, et al. Behaviors of adult *Agrilus planipennis* (Coleoptera: Buprestidae). *Gt Lakes Entomol* (2007) 40. Available at: https://scholar.valpo.edu/tgle/vol40/iss1/1/
16. Abell KJ, Duan JJ, Shrewsbury PM. Determining optimal parasitoid release timing for the biological control of emerald ash borer (Coleoptera: Buprestidae). *Florida Entomol* (2019) 102:691–94. doi: 10.1653/024.102.0403
17. Tluczek, A. R., D. G. McCullough, and T. M. Poland. Influence of host stress on emerald ash borer (Coleoptera: Buprestidae) adult density, development, and distribution in *Fraxinus pennsylvanica* trees. *Environ Entomol* (2011) 40:357–66. doi: 10.1603/EN10219

**Table S5.** Summary of locations and years for observations used to evaluate predictive accuracy of phenological events produced by the DDRP model for emerald ash borer.

| **Country** | **State** | **City** | **Lat** | **Lon** | **Year** | **Event(s)** |
| --- | --- | --- | --- | --- | --- | --- |
| United States | DC | DC | 38.906 | –76.973 | 2017 | peak adult activity |
| United States | DE | Newark | 39.668 | –75.742 | 2020 | first adult emergence, peak adult emergence |
| United States | GA | Suwanee | 34.027 | –84.049 | 2022 | first adult emergence |
| United States | LA | Andreas | 32.663 | –93.369 | 2016 | first adult emergence, peak adult emergence |
| United States | MI | Ann Arbor |  |  | 2003 | first adult emergence, peak adult emergence |
| United States | MI | Detroit |  |  | 2003 | peak adult emergence |
| United States | MI | Detroit |  |  | 2004 | peak adult emergence |
| United States | MI | Eastport | 45.114 | –85.332 | 2016 | first adult emergence, peak adult activity |
| United States | MI | Ithaca | 43.234 | –84.448 | 2016 | first adult emergence, peak adult activity |
| United States | MI | Ithaca | 43.234 | –84.448 | 2017 | first adult emergence, peak adult activity |
| United States | MI | Ithaca | 43.234 | –84.448 | 2018 | first adult emergence, peak adult activity |
| United States | MI | Ithaca | 43.234 | –84.448 | 2019 | first adult emergence, peak adult activity |
| United States | MI | Lansing | 42.808 | –84.359 | 2006 | peak adult activity |
| United States | MI | Lansing | 42.808 | –84.359 | 2007 | peak adult activity |
| United States | MI | Lansing | 42.694 | –84.382 | 2016 | first adult emergence, peak adult activity |
| United States | MI | Lansing | 42.694 | –84.382 | 2017 | first adult emergence, peak adult activity |
| United States | MI | Lansing | 42.694 | –84.382 | 2018 | first adult emergence, peak adult activity |
| United States | MI | Lansing | 42.694 | –84.382 | 2019 | first adult emergence, peak adult activity |
| United States | MI | Lansing | 42.697 | –84.375 | 2016 | first adult emergence, peak adult activity |
| United States | MI | Lansing | 42.697 | –84.375 | 2017 | first adult emergence, peak adult activity |
| United States | MI | Lansing | 42.697 | –84.375 | 2018 | first adult emergence, peak adult activity |
| United States | MI | Lansing | 42.697 | –84.375 | 2019 | first adult emergence, peak adult activity |
| United States | MI | Novi |  |  | 2004 | first adult emergence, peak adult emergence |
| United States | MI | Troy | 42.603 | –83.251 | 2020 | first adult emergence |
| United States | MI | Troy | 42.603 | –83.251 | 2021 | first adult emergence |
| United States | MI | Troy | 42.603 | –83.251 | 2022 | first adult emergence |
| United States | MI | Troy |  |  | 2004 | first adult emergence, peak adult emergence |
| United States | MI | Whitmore | 42.381 | –83.763 | 2005 | peak adult emergence |
| United States | NC | Garner | 35.644 | –78.58 | 2020 | first pupation |
| United States | NC | Garner | 35.644 | –78.58 | 2021 | first adult emergence, first pupation |
| United States | NC | Granville | 36.153 | –78.768 | 2017 | first pupation |
| United States | NY | Randolph | 42.162 | –78.975 | 2010 | first adult emergence |
| United States | NY | Rockland | 41.98 | –74.706 | 2021 | first adult emergence |
| United States | NY | Syracuse | 43.065 | –76.044 | 2016 | first adult emergence |
| United States | NY | Syracuse | 43.065 | –76.044 | 2017 | first adult emergence |
| United States | TN | Oak Ridge | 35.932 | –84.31 | 2017 | first egg hatch |
| United States | TN | Powell | 36.058 | –84.056 | 2016 | first egg hatch |
| United States | TN | Powell | 36.058 | –84.056 | 2017 | first egg hatch |
| United States | WI | Milwaukee | 42.986 | –88.184 | 2019 | first adult emergence |
| Russia | RU | Moscow | 55.983 | 37.167 | 2013 | first adult emergence |
| Russia | RU | Moscow | 55.983 | 37.167 | 2014 | first adult emergence |

**Table S6.** The start of development in degree-day units for each life stage of emerald ash borer according to the DDRP model that applied seven cohorts. Stages are presented in order of occurrence after overwintering J-larvae complete development (i.e., pupa, adult, egg, and larva). Individuals within each cohort are assumed to develop in synchrony.

| **Cohort** | **Perc. of population** | **Pupa** | **Adult** | **Egg** | **Larva** |
| --- | --- | --- | --- | --- | --- |
| 1 | 16.9 | 82 | 217 | 389 | 1089 |
| 2 | 22.4 | 123 | 258 | 430 | 1130 |
| 3 | 20 | 163 | 298 | 470 | 1170 |
| 4 | 15.5 | 204 | 339 | 511 | 1211 |
| 5 | 11 | 244 | 379 | 551 | 1251 |
| 6 | 7.7 | 285 | 420 | 592 | 1292 |
| 7 | 5.3 | 325 | 460 | 632 | 1332 |

## Supplementary Figures

**Figure S1**. Tables and corresponding plots of development rate, lower developmental threshold (Tlow), and duration in degree-days (DDs) estimated for oviposition longevity in emerald ash borer with and without the use of a forced x-intercept method.

I) No forcing

|  | **Temp (°C)** | **Days** | **1/days** |  |
| --- | --- | --- | --- | --- |
|  | 19.6 | 47.2 | 0.0212 | 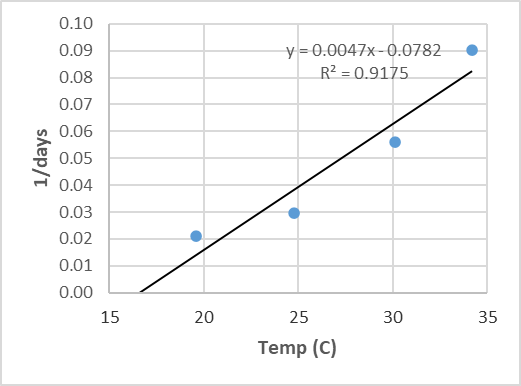 |
|  | 24.8 | 33.7 | 0.0297 |  |
|  | 30.1 | 17.8 | 0.0561 |  |
|  | 34 | 11.1 | 0.0903 |  |
|  |  |  |  |  |
| Slope | (b) |  | 0.0047 |  |
| Intercept | (a) |  | −0.0782 |  |
| R^2^ |  |  | 0.918 |  |
| Tlow (°C) | (−a/b) |  | 16.7 |  |
| Tlow (°F) | (−a/b) |  | 62.1 |  |
| DDs (°C) | 1/slope |  | 213 |  |
| DDs (°F) | 1/slope |  | 383 |  |
|  |  |  |  |  |

II) With forcing

|  | **Temp (°C)** | **Days** | **1/days** |  |
| --- | --- | --- | --- | --- |
|  | 12.2 |  | 0.0099 | 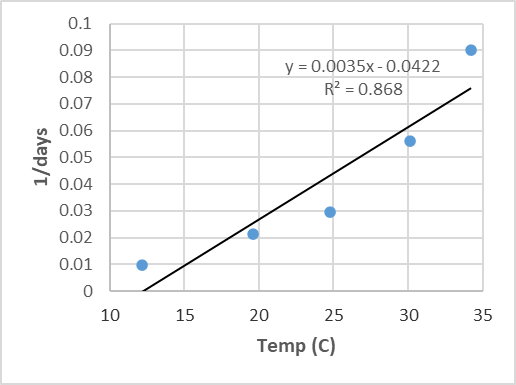 |
|  | 19.6 | 47.2 | 0.0212 |  |
|  | 24.8 | 33.7 | 0.0297 |  |
|  | 30.1 | 17.8 | 0.0561 |  |
|  | 34 | 11.1 | 0.0903 |  |
|  |  |  |  |  |
| Slope | (b) |  | 0.0035 |  |
| Intercept | (a) |  | −0.0422 |  |
| R^2^ |  |  | 0.868 |  |
| Tlow (°C) | (−a/b) |  | 12.2 |  |
| Tlow (°F) | (−a/b) |  | 54.0 |  |
| DDs (°C) | 1/slope |  | 289 |  |
| DDs (°F) | 1/slope |  | 522 |  |
|  |  |  |  |  |

**Figure S2**. Tables and corresponding plots of development rate, lower developmental threshold (Tlow), and duration in degree-days (DDs) estimated for the egg stage in emerald ash borer with and without the use of a forced x-intercept method.

I) No forcing

|  | **Temp (°C)** | **Days** | **1/days** |  |
| --- | --- | --- | --- | --- |
|  | 20 | 20 | 0.0500 | 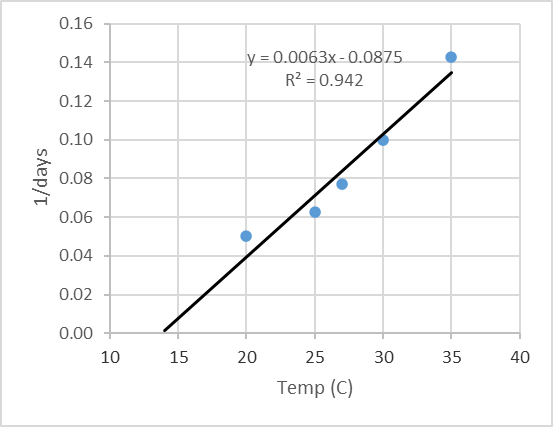 |
|  | 25 | 16 | 0.0625 |  |
|  | 27 | 13 | 0.0769 |  |
|  | 30 | 10 | 0.1000 |  |
|  | 35 | 7 | 0.1429 |  |
|  |  |  |  |  |
| Slope | (b) |  | 0.0063 |  |
| Intercept | (a) |  | −0.0875 |  |
| R^2^ |  |  | 0.942 |  |
| Tlow (°C) | (−a/b) |  | 13.8 |  |
| Tlow (°F) | (−a/b) |  | 56.8 |  |
| DDs (°C) | 1/slope |  | 159 |  |
| DDs (°F) | 1/slope |  | 283 |  |
|  |  |  |  |  |

II) With forcing

|  | **Temp (°C)** | **Days** | **1/days** |  |
| --- | --- | --- | --- | --- |
|  | 12.2 |  | 0.0049 |  |
|  | 20 | 20 | 0.0500 | 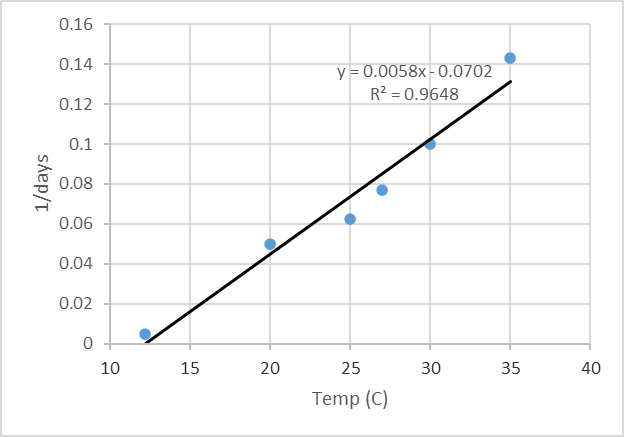 |
|  | 25 | 16 | 0.0625 |  |
|  | 27 | 13 | 0.0769 |  |
|  | 30 | 10 | 0.1000 |  |
|  | 35 | 7 | 0.1429 |  |
|  |  |  |  |  |
| Slope | (b) |  | 0.0058 |  |
| Intercept | (a) |  | −0.0702 |  |
| R^2^ |  |  | 0.9648 |  |
| Tlow (°C) | (−a/b) |  | 12.2 |  |
| Tlow (°F) | (−a/b) |  | 54 |  |
| DDs (°C) | 1/slope |  | 174 |  |
| DDs (°F) | 1/slope |  | 313 |  |
|  |  |  |  |  |

**Figure S3**. Table and corresponding plot of development rate, lower developmental threshold (Tlow), and duration in degree-days (DDs) estimated for larval instars 1−4 (egg hatch to formation of J-larvae) in emerald ash borer with the use of a forced x-intercept method.

|  | **Temp (°C)** | **Days** | **1/days** |  |
| --- | --- | --- | --- | --- |
|  | 12.5 |  | 0.0006 | 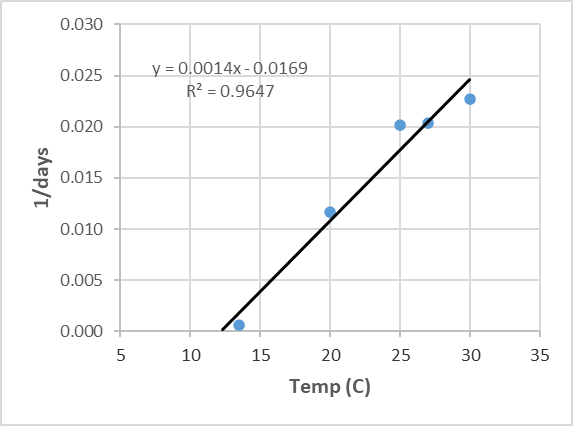 |
|  | 20 | 66 | 0.0151 |  |
|  | 25 | 34 | 0.0297 |  |
|  | 27 | 36 | 0.0278 |  |
|  |  |  |  |  |
| Slope | (b) |  | 0.0021 |  |
| Intercept | (a) |  | −0.0251 |  |
| R^2^ |  |  | 0.9648 |  |
| Tlow (°C) | (−a/b) |  | 12.2 |  |
| Tlow (°F) | (−a/b) |  | 54 |  |
| DDs (°C) | 1/slope |  | 488 |  |
| DDs (°F) | 1/slope |  | 878 |  |
|  |  |  |  |  |

**Figure S4**. Line plots of adult emergence events in emerald ash borer vs. degree-day accumulation [lower developmental threshold (Tlow) = 12.2 °C] at six locations in 2011 and 2012. Shapes depict the timing of first, 50%, and 95% adult emergence (circle, triangle, and square, respectively) for each location. Gray vertical lines depict the predicted timing of adult emergence for each of seven cohorts according to the final DDRP model. The first, fourth, and last lines are emphasized with dark gray and labels to emphasize the model-predicted timing of first, 50% (peak), and last adult emergence.


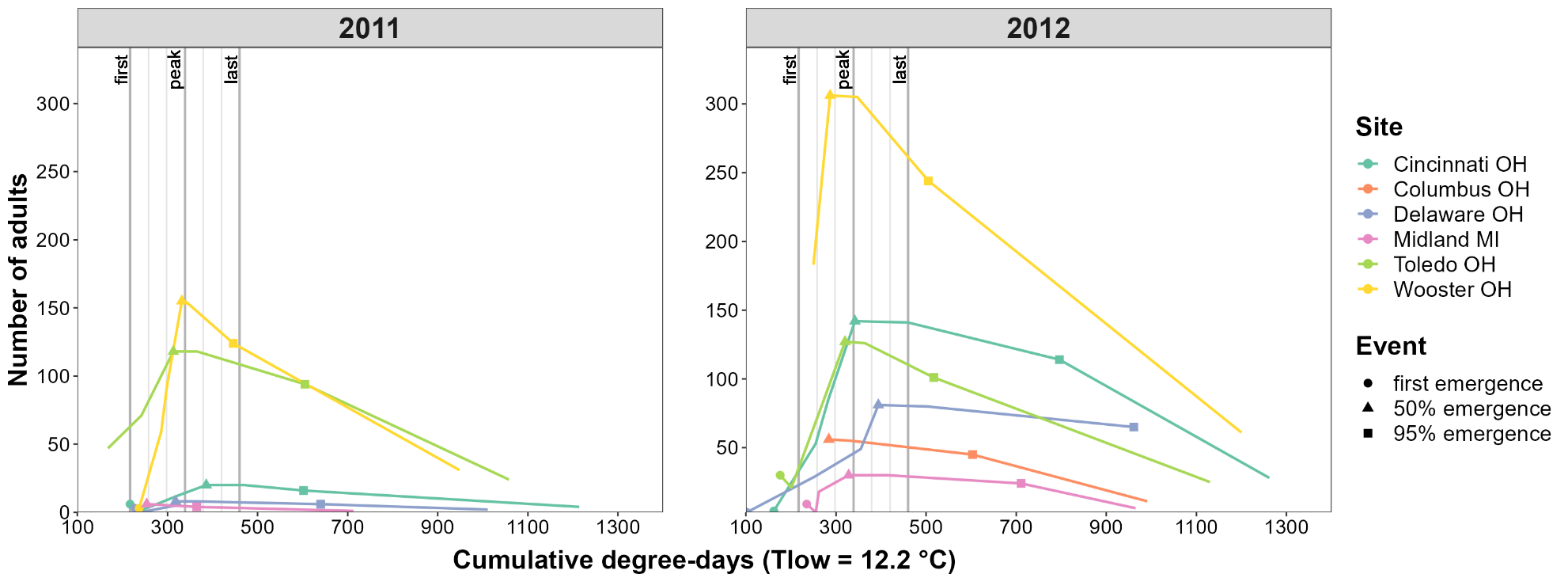


**Figure S5.** Maps depicting cold days and model-predicted cold stress accumulation for emerald ash borer in China. Maps of (a) consecutive days of minimum temperatures (*T_min_*) below ‒31 °C based on 20-year climate averages centered on 2008 (1999−2018, northeastern China only), (b) annual cold stress accumulation based on 20-year climate averages, and (c) annual cold stress accumulation based on climate data for an extreme year in terms of cold stress accumulation (2001) were used to calibrate moderate and severe cold stress limits in the DDRP model. Maps (b) and (c) were produced using the final DDRP model.

**
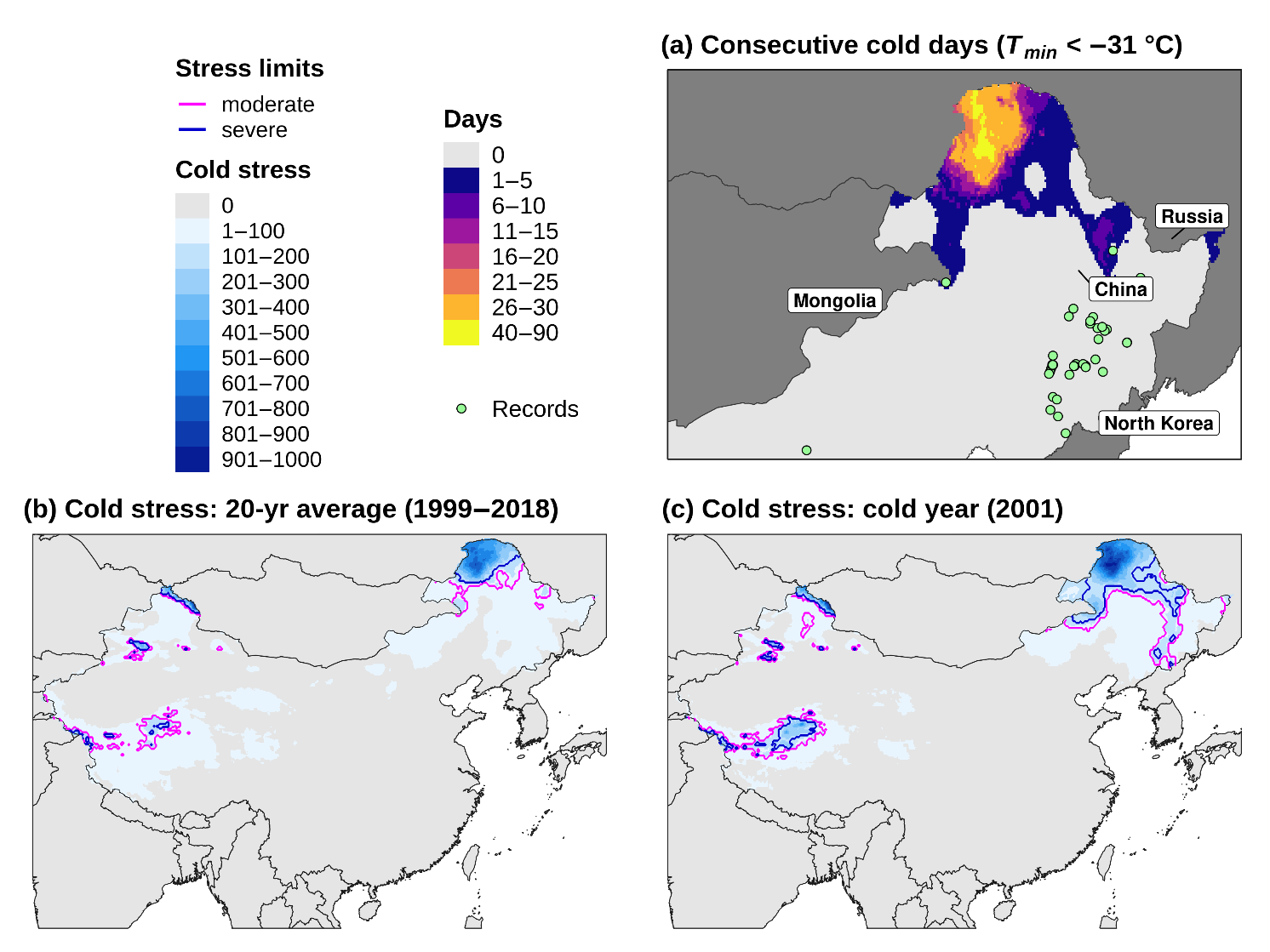
**

**Figure S6.** Maps depicting heat days and model-predicted heat stress accumulation for emerald ash borer in the southern United States and Mexico. Maps of (a) consecutive days of maximum temperatures (*T_max_*) above 38 °C based on 20-year climate averages centered on 2011 (2002−2021), (b) annual heat stress accumulation based on 20-year climate averages, and (c) annual heat stress accumulation based on climate data for an extreme year in terms of heat stress accumulation (2011) were used to calibrate moderate and severe heat stress limits in the DDRP model. Maps (b) and (c) were produced using the final DDRP model.


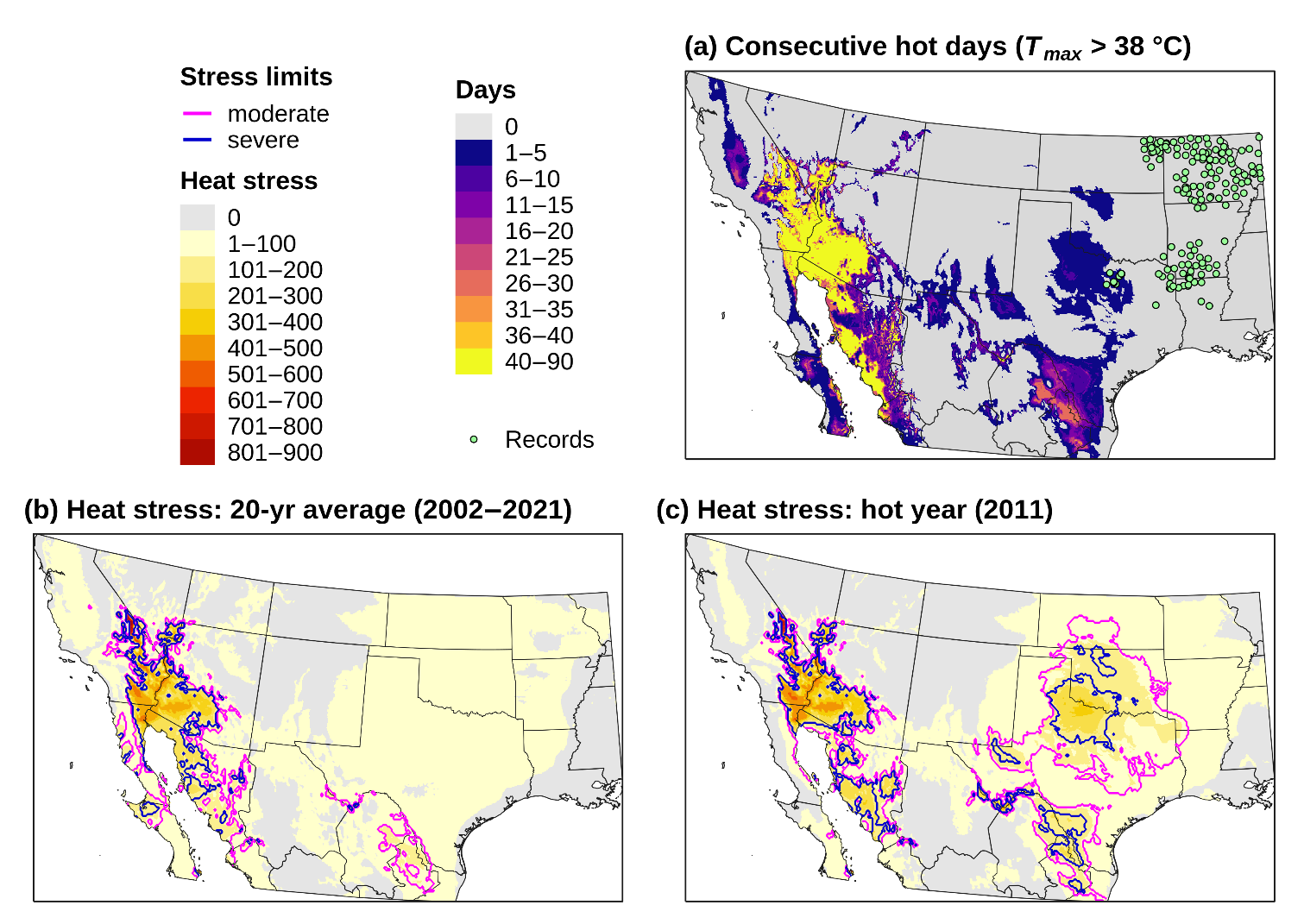

Supplement: Supplementary file 1 [file DataSheet_1.docx]
